# Endosomal trafficking protein TBC‐2 modulates stress resistance and lifespan through DAF‐16‐dependent and independent mechanisms

**DOI:** 10.1111/acel.13762

**Published:** 2023-02-15

**Authors:** Annika Traa, Sonja K. Soo, Abdelrahman AlOkda, Bokang Ko, Christian E. Rocheleau, Jeremy M. Van Raamsdonk

**Affiliations:** ^1^ Department of Neurology and Neurosurgery McGill University Montreal Quebec Canada; ^2^ Metabolic Disorders and Complications Program Research Institute of the McGill University Health Centre Montreal Quebec Canada; ^3^ Brain Repair and Integrative Neuroscience Program Research Institute of the McGill University Health Centre Montreal Quebec Canada; ^4^ Division of Experimental Medicine Department of Medicine McGill University Montreal Quebec Canada

**Keywords:** aging, *C. elegans*, DAF‐16/FOXO, endosomal trafficking, genetics, lifespan, stress resistance, TBC‐2

## Abstract

The FOXO transcription factor, DAF‐16, plays an integral role in insulin/IGF‐1 signaling (IIS) and stress response. In conditions of stress or decreased IIS, DAF‐16 moves to the nucleus where it activates genes that promote survival. To gain insight into the role of endosomal trafficking in resistance to stress, we disrupted *tbc‐2*, which encodes a GTPase activating protein that inhibits RAB‐5 and RAB‐7. We found that *tbc‐2* mutants have decreased nuclear localization of DAF‐16 in response to heat stress, anoxia, and bacterial pathogen stress, but increased nuclear localization of DAF‐16 in response to chronic oxidative stress and osmotic stress. *tbc‐2* mutants also exhibit decreased upregulation of DAF‐16 target genes in response to stress. To determine whether the rate of nuclear localization of DAF‐16 affected stress resistance in these animals, we examined survival after exposure to multiple exogenous stressors. Disruption of *tbc‐2* decreased resistance to heat stress, anoxia, and bacterial pathogen stress in both wild‐type worms and stress‐resistant *daf‐2* insulin/IGF‐1 receptor mutants. Similarly, deletion of *tbc‐2* decreases lifespan in both wild‐type worms and *daf‐2* mutants. When DAF‐16 is absent, the loss of *tbc‐2* is still able to decrease lifespan but has little or no impact on resistance to most stresses. Combined, this suggests that disruption of *tbc‐2* affects lifespan through both DAF‐16‐dependent and DAF‐16‐independent pathways, while the effect of *tbc‐2* deletion on resistance to stress is primarily DAF‐16‐dependent. Overall, this work demonstrates the importance of endosomal trafficking for the proper nuclear localization of DAF‐16 during stress and that perturbation of normal endosomal trafficking is sufficient to decrease both stress resistance and lifespan.

## Graphical abstract

Disruption of the endosomal trafficking protein TBC‐2 alters the nuclear localization of the FOXO transcription factor DAF‐16 in response to stress. This results in decreased upregulation of stress response genes, decreased resistance to multiple stresses, and decreased lifespan. This work highlights the importance of endosomal trafficking for stress resistance and lifespan through its effects on stress response signaling pathways.

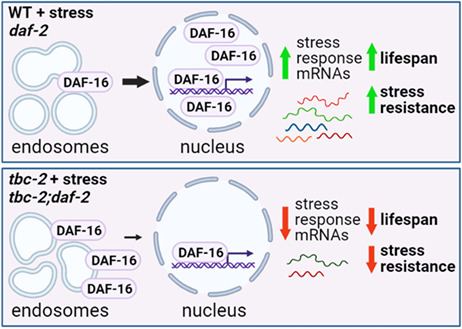

**Abbreviations**
ANOVA: analysis of variance
cDNA: complementary deoxyribonucleic acid
ER: endoplasmic reticulum
FUdR: fluorodeoxyuridine
GAP: GTPase activating protein
GFP: green fluorescent protein
GTP: guanosine triphosphate
IGF‐1: insulin growth factor 1
IIS: insulin/IGF‐1 signaling
NGM: nematode growth medium
mRNA: messenger ribonucleic acid
qPCR: quantitative polymerase chain reaction
RNAi: RNA interference
RT‐PCR: reverse transcriptase polymerase chain reaction
SEM: standard error of the mean

## 1 INTRODUCTION

The insulin/IGF‐1 signaling (IIS) pathway is a growth factor signaling pathway that has an evolutionarily conserved effect on lifespan (Kenyon, 2005), which was first demonstrated in the worm *C. elegans* (Friedman & Johnson, 1988; Kenyon et al., 1993). Genes from this pathway were among the first genes shown to affect longevity. This includes *daf‐2*, which encodes the insulin/IGF‐1 receptor (Kenyon et al., 1993) and *age‐1*, which encodes phosphatidylinositol 3‐kinase (Friedman & Johnson, 1988). The ability of *daf‐2* and *age‐1* mutations to extend lifespan is completely dependent on the FOXO transcription factor DAF‐16 (Lin et al., 1997; Ogg et al., 1997). Both the insulin/IGF‐1 receptor and phosphatidylinositol 3‐kinase act upstream of DAF‐16, preventing its translocation to the nucleus when IIS levels are normal and in the absence of stress. Under conditions of decreased IIS, or in *daf‐2* and *age‐1* mutants, DAF‐16 moves from the cytoplasm to the nucleus to induce changes in gene expression, including the upregulation of genes involved in stress response and metabolism. The loss of DAF‐16 has also been shown to decrease the lifespan of multiple other long‐lived mutants including long‐lived mitochondrial mutants (*clk‐1*, *isp‐1*, *nuo‐6*; Senchuk et al., 2018), germline ablation mutants (*glp‐1*; Berman & Kenyon, 2006; Chen et al., 2015), dietary restriction mutants (*eat‐2*; Lakowski & Hekimi, 1998), chemosensory mutants (*osm‐5*; Apfeld & Kenyon, 1999), and mutants with decreased translation (*ife‐2*; Hansen et al., 2007; Syntichaki et al., 2007).

In addition to its role in longevity, DAF‐16 is important for the survival of exogenous stressors. Multiple external stressors cause nuclear localization of DAF‐16 including heat stress, anoxia, oxidative stress, osmotic stress, starvation, and exposure to bacterial pathogens (Dues et al., 2016; Henderson & Johnson, 2001). In the nucleus, DAF‐16 causes the upregulation of genes that help to protect against external stressors such as superoxide dismutase (Murphy et al., 2003; Tepper et al., 2013). As a result, disruption of *daf‐16* leads to decreased stress resistance in wild‐type worms (Lin et al., 2018). Conversely, long‐lived *daf‐2* mutants, which have increased nuclear localization of DAF‐16, exhibit increased resistance to multiple external stressors including oxidative stress, heat stress, osmotic stress, anoxia, heavy metals, and bacterial pathogens (Barsyte et al., 2001; Dues et al., 2019; Garsin et al., 2003; Honda & Honda, 1999; Lithgow et al., 1995). The enhanced resistance to stress in *daf‐2* mutants is mediated by DAF‐16 (Dues et al., 2019).

We recently elucidated a crucial role for endosomal trafficking in the proper localization of DAF‐16 within the cell (Meraş et al., 2022). Specifically, we showed that DAF‐16 can be localized to endosomes and that this endosomal localization is increased by IIS. Combined with previous studies demonstrating that multiple components of the IIS pathway can localize to endosomes (Bergeron et al., 2016; Naguib et al., 2015; Schenck et al., 2008; Shinde & Maddika, 2016; Walz et al., 2010), this suggests the possibility that IIS takes place on endosomes.

In our previous work, we found that the localization of DAF‐16 to endosomes can be modulated by disrupting proteins involved in endosome trafficking (Meraş et al., 2022). During endosomal trafficking, internalized vesicles containing receptors and bound ligands undergo homotypic fusion to form early endosomes, which contain RAB‐5. After sorting at the early endosome, internalized cargoes can be recycled back to the plasma membrane via recycling endosomes, transported to the Trans‐Golgi network, or degraded by the lysosome after trafficking through late endosomes, which contain RAB‐7.

The endosomal trafficking protein TBC‐2 is a GTPase activating protein (GAP) that functions to inactivate RAB‐5 during endosome maturation (Chotard et al., 2010; Law & Rocheleau, 2017). TBC‐2 can also inhibit RAB‐7 (Chotard et al., 2010; Law & Rocheleau, 2017) and has a role in endosomal recycling through its interaction with AMPH‐1 and RAB‐10 (Liu & Grant, 2015). Disruption of *tbc‐2* results in an accumulation of enlarged late endosomes, which is phenocopied by constitutive activation of RAB‐5 (Chotard et al., 2010).

Disruption of *tbc‐2* results in increased endosomal localization of DAF‐16, while genetic knockdown of the RAB‐5 or RAB‐7 GTPases, which are inactivated by TBC‐2's GAP activity, decreases the endosomal localization of DAF‐16 (Meraş et al., 2022). Disruption of *tbc‐2* significantly decreases the lifespan of long‐lived *daf‐2* mutants.

Based on the ability of *tbc‐2* to modulate the localization of DAF‐16 within the cell and the importance of nuclear localization of DAF‐16 for surviving external stressors, we explored the role of TBC‐2 in responding to stress. We found that disruption of *tbc‐2* alters the nuclear localization of DAF‐16 in response to stress thereby affecting worms' ability to enact stress‐responsive changes in gene expression. *tbc‐2* mutants exhibit decreased resistance to multiple stresses and the loss of *tbc‐2* decreases resistance to stress in stress‐resistant *daf‐2* mutants. Interestingly, the disruption of *tbc‐2* can affect lifespan in wild‐type and *daf‐2* worms lacking *daf‐16*, indicating that TBC‐2 can have DAF‐16‐dependent and DAF‐16‐independent effects on longevity. Overall, this work highlights the importance of endosomal trafficking in stress resistance and lifespan.

## 2 RESULTS

### 2.1 TBC‐2 is required for proper nuclear localization of DAF‐16 during stress

Under unstressed conditions, disruption of *tbc‐2* increases the localization of DAF‐16 to endosomes (Meraş et al., 2022). As the nuclear localization of DAF‐16 is important for responding to stress and for longevity, we wondered whether the increase in endosomal localization would affect the ability of DAF‐16 to move to the nucleus in response to exogenous stressors. Accordingly, we used a DAF‐16 translational fusion strain in which DAF‐16 tagged with GFP is expressed under the endogenous *daf‐16* promoter (*zIs356[daf‐16p::daf‐16::GFP]*) to examine the nuclear localization of DAF‐16 in response to stress in a wild‐type and *tbc‐2* mutant background (Henderson & Johnson, 2001). We utilized stress paradigms that we previously found to result in nuclear localization of DAF‐16 (Dues et al., 2016).

It is important to note that different stresses induce the nuclear localization of DAF‐16 to different extents. For example, heat stress and anoxia result in very obvious nuclear localization of DAF‐16, while some stresses, like cold stress or endoplasmic reticulum stress, result in little or no nuclear localization of DAF‐16 (Dues et al., 2016). As a result, it was not possible to have an identical baseline of DAF‐16 nuclear localization across all of the stresses we examined because DAF‐16 reacts differently to each type of stress. In addition, each stress causes a different pattern of DAF‐16 nuclear localization with respect to which tissues exhibit the greatest nuclear localization of DAF‐16. For both of these reasons, it is not possible to directly compare nuclear localization between stresses. Instead, we focused on differences between wild‐type and *tbc‐2* mutants for each stress individually.

**FIGURE 1:**
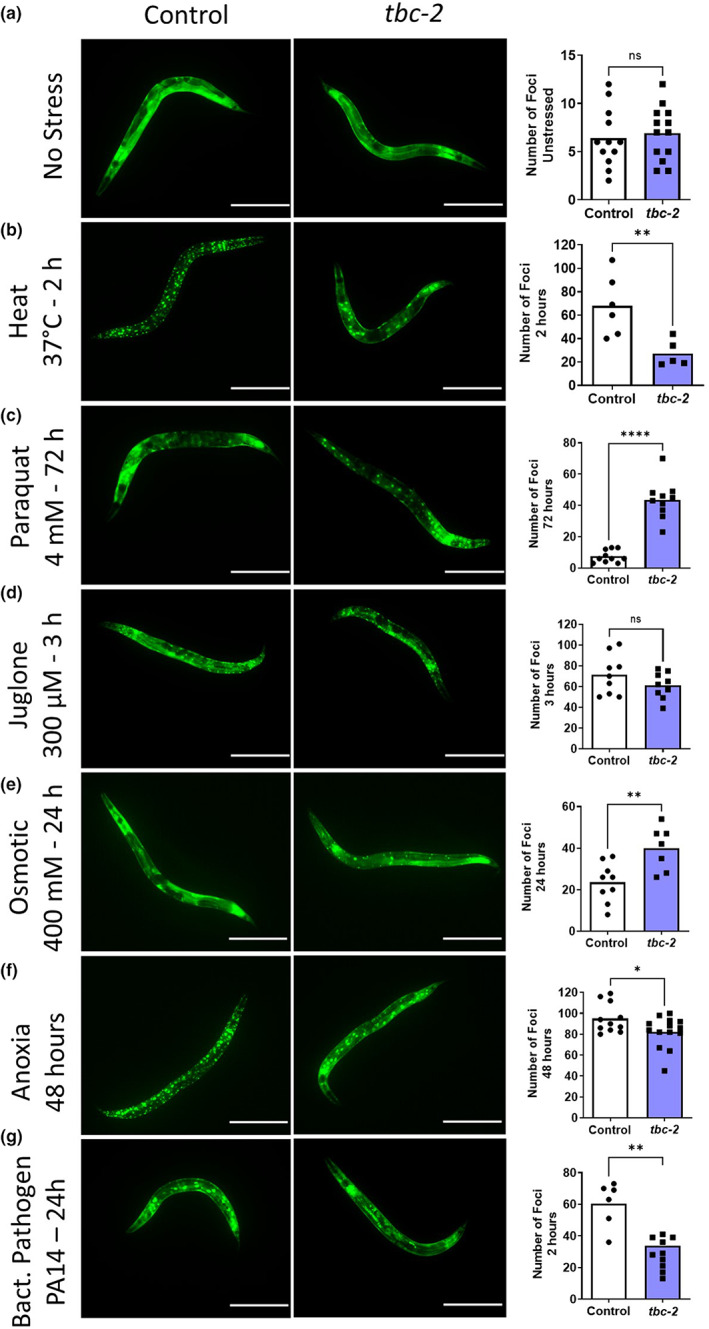
Disruption of TBC‐2 alters nuclear localization of DAF‐16 in response to stress. The effect of *tbc‐2* deletion on the nuclear localization of DAF‐16 in response to stress was examined using the translational fusion strain: *zIs356*[*daf‐16p*::*daf‐16::GFP*]. In absence of stress, DAF‐16 is primarily localized to the cytoplasm in both wild‐type and *tbc‐2* worms (a). After exposure to heat stress (37°C, 2 h), *tbc‐2* mutants show decreased nuclear localization of DAF‐16 compared with wild‐type worms (b). After exposure to chronic oxidative stress (4 mM paraquat, 72 h), *tbc‐2* mutants exhibit increased nuclear localization of DAF‐16 compared with wild‐type worms (c). *tbc‐2* deletion does not affect the nuclear localization of DAF‐16 in response to acute oxidative stress (300 μM juglone, 3 h) (d). Disruption of *tbc‐2* increases the nuclear localization of DAF‐16 after osmotic stress (400 mM NaCl, 24 h) (e). After anoxia (48 h), *tbc‐2* mutants have decreased nuclear localization of DAF‐16 compared with wild‐type worms (f). *tbc‐2* deletion mutants have decreased nuclear localization of DAF‐16 after exposure to bacterial pathogens (*P. aeruginosa* strain PA14, 24 h) (g). Overall, disruption of *tbc‐2* significantly impacts the nuclear localization of DAF‐16 in response to external stressors. Scale bars indicate 300 μm. Three biological replicates were performed. Statistical significance was assessed using a *t* test. *p < 0.05, **p < 0.01, ****p < 0.0001

Under unstressed conditions, DAF‐16 is primarily localized to the cytoplasm in wild‐type and *tbc‐2* mutants (Figure 1a). After exposure to 37°C heat stress for 2 h, *tbc‐2* worms showed decreased nuclear localization of DAF‐16 compared with wild‐type animals (Figure 1b). In contrast, loss of *tbc‐2* increased the nuclear localization of DAF‐16 during chronic oxidative stress (4 mM paraquat, 3 days; Figure 1c). After exposure to acute oxidative stress (300 μM juglone, 3 h), DAF‐16 nuclear localization did not differ between wild‐type and *tbc‐2* mutants (Figure 1d). Nuclear localization of DAF‐16 was increased in *tbc‐2* mutants compared with wild‐type worms after exposure to osmotic stress (400 mM NaCl, 24 h; Figure 1e), but decreased compared with wild‐type worms after exposure to anoxia (48 h; Figure 1f) or bacterial pathogen stress (*P. aeruginosa* strain PA14, 24 h; Figure 1g). Overall, deletion of *tbc‐2* disrupts the nuclear localization of DAF‐16 in response to exogenous stressors with the direction of modulation varying by external stressor.

To ensure that the differences that we observed in nuclear localization of DAF‐16 were not the result of the specific time point that we examined, we performed a time course of nuclear localization during heat stress, chronic oxidative stress, acute oxidative stress, and anoxia. During exposure to 37°C heat stress, nuclear localization of DAF‐16 was significantly decreased in *tbc‐2* mutants at 2 and 4 h (Figures S1 and S2a). Chronic oxidative stress from 4 mM paraquat resulted in minimal nuclear localization of DAF‐16 in a wild‐type background at 1, 2, or 3 days. At the 3‐day time point, *tbc‐2* mutants exhibited a significant increase in DAF‐16 nuclear localization compared with wild‐type worms (Figure S2b). In contrast, there was no difference in nuclear localization of DAF‐16 at any time point during exposure to acute oxidative stress resulting from 300 μM juglone (Figure S2c). We also did not observe any difference in nuclear localization of DAF‐16 after exposure to anoxic stress at any time point examined (Figure S2d).

To determine whether the difference we observed between chronic and acute oxidative stress resulted from the chronic or acute nature of the assay, we examined the effect of acute exposure to a higher dose of paraquat on nuclear localization of DAF‐16. We have previously shown that a high dose of paraquat results in a rapid decrease in survival, similar to juglone (Schaar et al., 2015). As with acute exposure to juglone, we found that there were no significant differences in the nuclear localization of DAF‐16 following acute exposure to 220 mM paraquat (Figure S3).

### 2.2 TBC‐2 is required for DAF‐16 to upregulate stress response genes after exposure to exogenous stressors

Since disruption of *tbc‐2* alters the nuclear localization of DAF‐16 in response to stress, we wondered whether this would affect the ability of DAF‐16 to upregulate stress‐responsive target genes. To examine the role of *tbc‐2* in DAF‐16‐mediated gene expression changes in response to stress, we exposed wild‐type and *tbc‐2* mutant worms to the same external stressors that we used to examine DAF‐16 nuclear localization and then quantified the expression of six different DAF‐16 target genes using quantitative RT‐PCR. These genes included *sod‐3*, *dod‐3*, *mtl‐1*, *sodh‐1*, *ftn‐1*, and *icl‐1* (Senchuk et al., 2018; Tepper et al., 2013).

**FIGURE 2:**
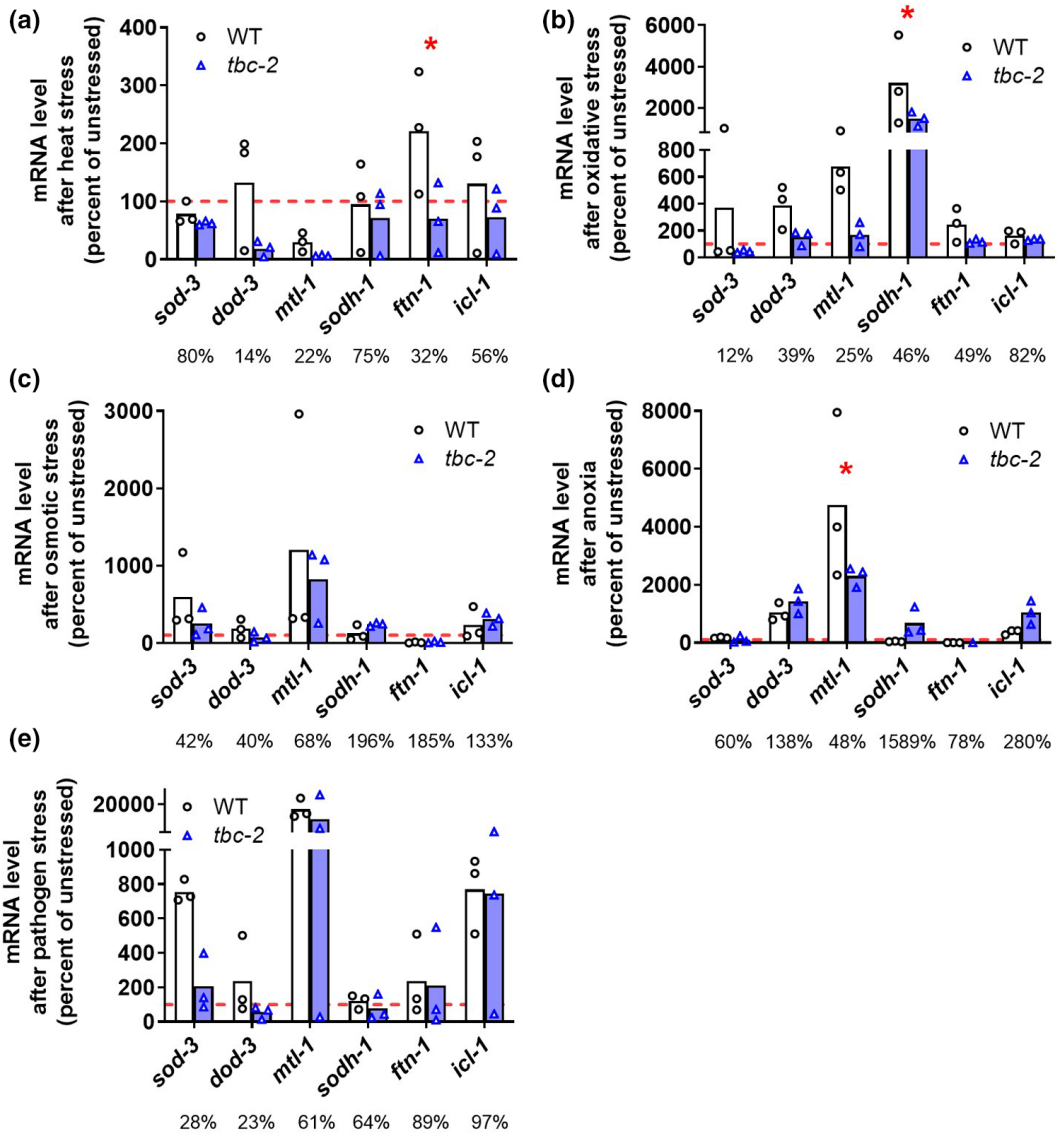
*tbc‐2* mutants have decreased upregulation of DAF‐16 target genes in response to stress. Wild‐type worms and *tbc‐2* mutants were exposed to five different exogenous stressors to examine the upregulation of DAF‐16 target genes including *sod‐3*, *dod‐3*, *mtl‐1*, *sodh‐1*, *ftn‐1* and *icl‐1*. After exposure to heat stress (35°C, 2 h with 24‐h recovery), *ftn‐1* exhibited a significantly greater upregulation in wild‐type worms compared with *tbc‐2* mutants (a). After exposure to oxidative stress (4 mM paraquat, 48 h), *sodh‐1* showed a greater magnitude of upregulation in wild‐type worms than in *tbc‐2* worms (b). There were no significant differences in the upregulation of DAF‐16 target genes in response to osmotic stress (400 mM NaCl, 24 h) (c). After exposure to anoxia (48, 24‐h recovery), wild‐type worms exhibited a significantly greater upregulation of *mtl‐1* than *tbc‐2* mutants (d). The magnitude of upregulation of DAF‐16 target genes after exposure to bacterial pathogens (*P. aeruginosa* strain PA14, 24 h) was not significantly different between wild‐type worms and *tbc‐2* mutants (e). The percentages under each bar indicate the percentage increase in gene expression in *tbc‐2* mutants in response to stress as a percentage of the percentage increase in gene expression in wild‐type worms in response to stress. Overall, *tbc‐2* mutants exhibit a mild decrease in the upregulation of DAF‐16 target genes in response to exogenous stressors. Three biological replicates were performed. Statistical significance was assessed using a two‐way ANOVA with Šidák's multiple comparisons test. *p < 0.05

We found that with each of the five external stressors we examined, there was a trend toward decreased expression of DAF‐16 target genes in *tbc‐2* mutants compared with wild‐type animals (Figure 2). This trend was most obvious for heat stress, oxidative stress, and bacterial pathogen stress (Figure 2a,b,e). In most cases, the differences in gene expression that we observed failed to reach significance because of the high degree of variability in gene expression that occurs after exposure to stress. Nonetheless, for multiple stresses, there were specific DAF‐16 target genes that showed a significant decrease in upregulation in response to stress in *tbc‐2* mutants compared with wild‐type. Combined, these results suggest that TBC‐2 is required for the full upregulation of DAF‐16 target genes in response to stress.

### 2.3 TBC‐2 is required for the survival of exogenous stressors

**FIGURE 3:**
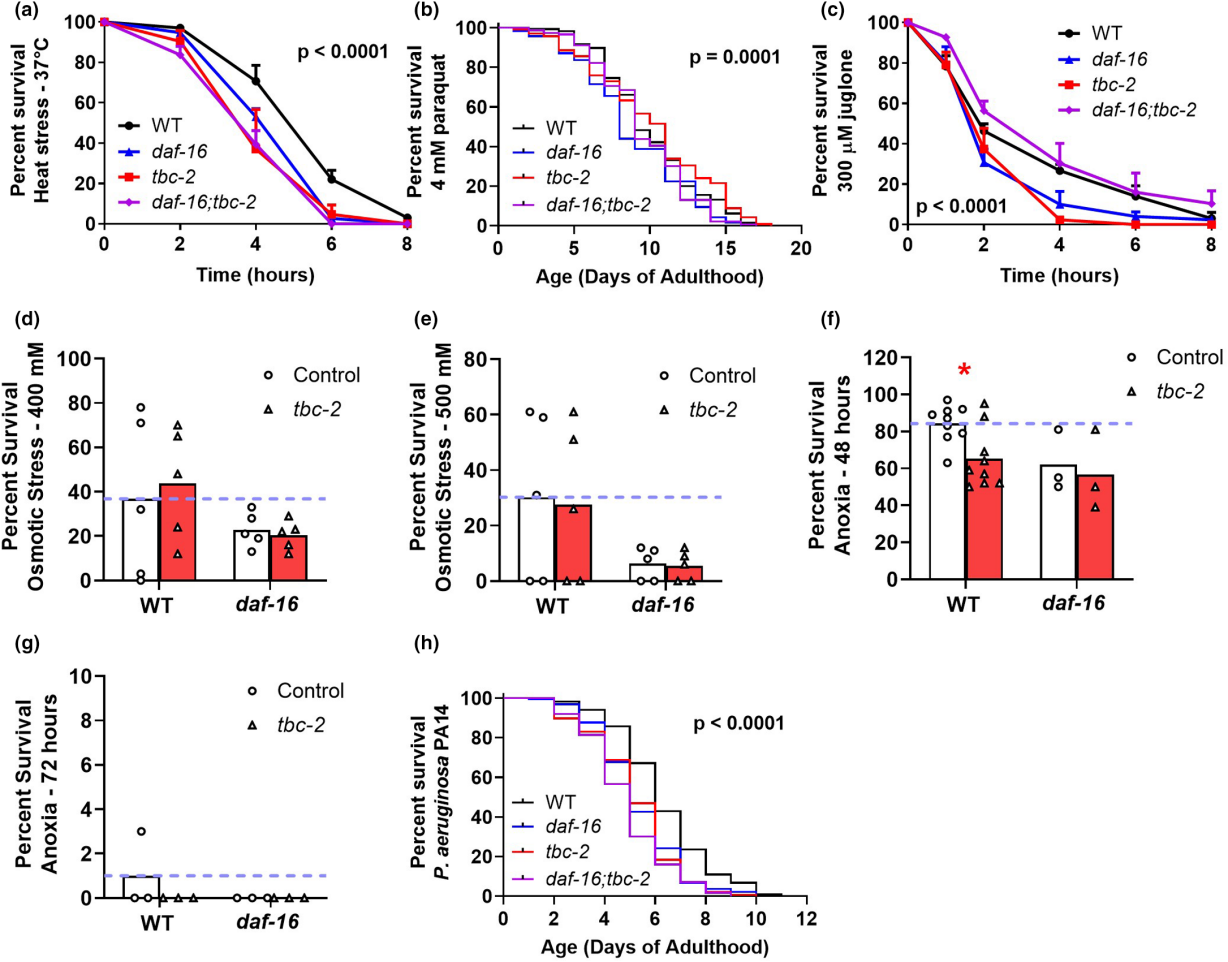
Disruption of TBC‐2 decreases resistance to external stressors. Deletion of *tbc‐2* decreases the survival of heat stress at 37°C (a) but does not significantly affect survival during chronic oxidative stress (4 mM paraquat) (b). *tbc‐2* mutants have decreased survival during acute oxidative stress (300 μM juglone) (c). Deletion of *tbc‐2* does not affect resistance to osmotic stress (400–500 mM NaCl) (d, e) but does reduce resistance to anoxia (48–72 h) (f, g). Disruption of *tbc‐2* also decreases resistance to bacterial pathogens (*P. aeruginosa* strain PA14) (h). Overall, *tbc‐2* mutants exhibit decreased resistance to multiple external stressors. To determine the extent to which *tbc‐2* can affect stress resistance independently of DAF‐16, the effect of *tbc‐2* deletion on stress resistance in *daf‐16* deletion mutants was examined. The stress resistance of *daf‐16;tbc‐2* mutants was not further decreased compared with *daf‐16* single mutants in any of the stress assays (a–h). A minimum of three biological replicates were performed. Statistical significance was assessed using the log‐rank test for panels (a), (b), (c), and (h). The overall *p*‐value for these panels is indicated. *p*‐values for comparisons between pairs of survival plots can be found in Table S1. Statistical significance was assessed using a two‐way ANOVA with Šidák's multiple comparison test for panels (d), (e), (f), and (g). *p < 0.05

Since DAF‐16 is important for responding to stress, we next sought to determine how the altered localization of DAF‐16 in response to *tbc‐2* deletion would affect the survival of exogenous stressors in wild‐type worms. Disruption of *tbc‐2* decreased resistance to heat stress (37°C; Figure 3a) but did not affect resistance to chronic oxidative stress (4 mM paraquat; Figure 3b). Deletion of *tbc‐2* decreased resistance to acute oxidative stress (300 μM juglone; Figure 3c) but had no effect on resistance to osmotic stress (400, 500 mM NaCl; Figure 3d,e). *tbc‐2* mutants have decreased resistance to anoxia (48, 72 h; Figure 3f,g) and bacterial pathogens (*P. aeruginosa* strain PA14, 24 h; Figure 3h) compared with wild‐type worms. Combined, these results indicate that disruption of *tbc‐2* results in decreased resistance to multiple external stressors. Notably, *tbc‐2* mutants had decreased resistance to all three stresses for which disruption of *tbc‐2* causes decreased nuclear localization of DAF‐16.

**FIGURE 4:**
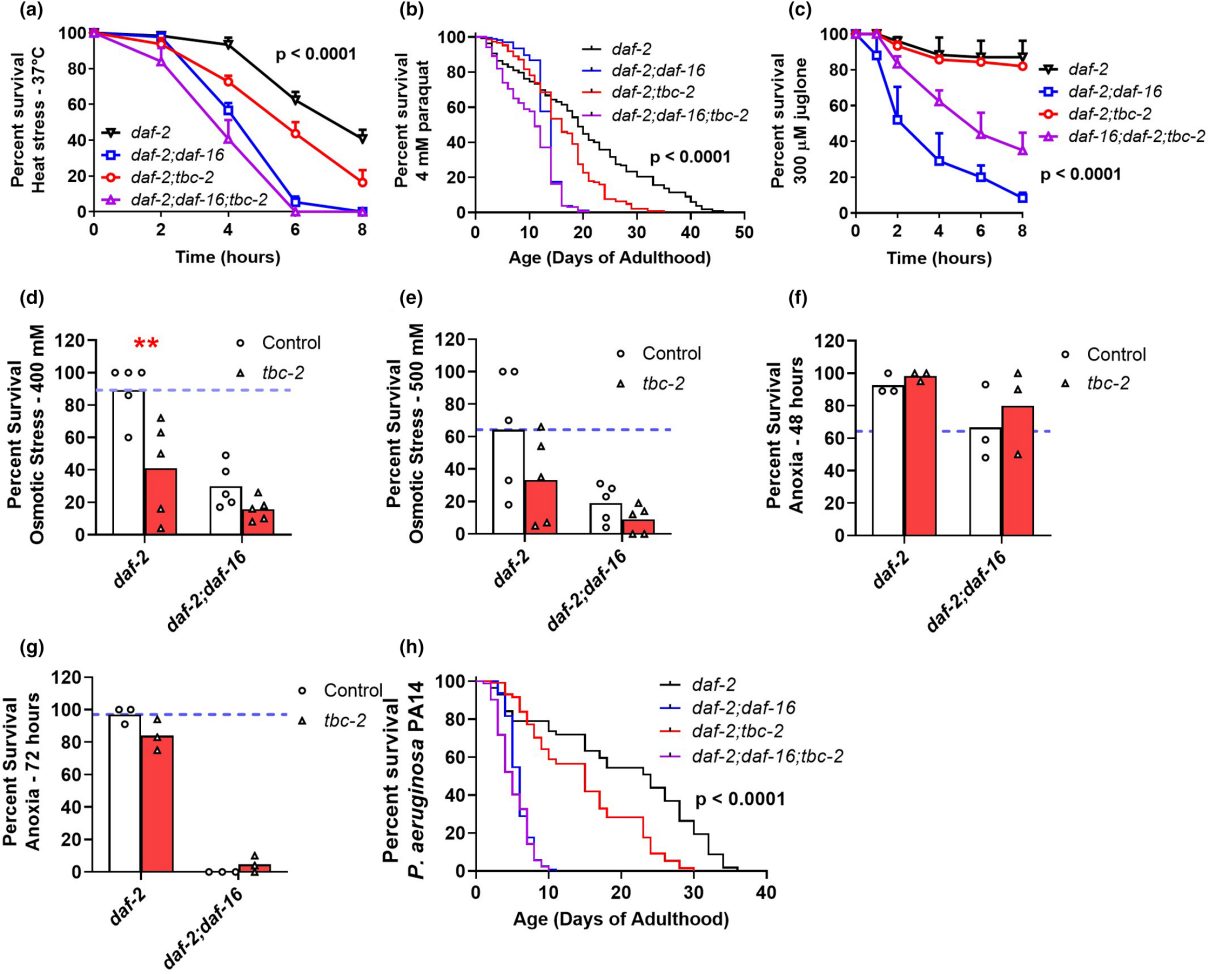
TBC‐2 is required for enhanced stress resistance of long‐lived *daf‐2* mutants. Disruption of *tbc‐2* decreased the survival of *daf‐2* worms exposed to heat stress at 37°C (a) and chronic oxidative stress (4 mM paraquat) (b). In contrast, loss of *tbc‐2* did not affect resistance to acute oxidative stress in *daf‐2* worms (300 μM juglone) (c). Deletion of *tbc‐2* decreased resistance to osmotic stress (400–500 mM NaCl) (d, e), anoxia (48–96 h) (f, g), and bacterial pathogens (*P. aeruginosa* strain PA14) (h) in *daf‐2* worms. To determine the extent to which *tbc‐2* can affect stress resistance independently of DAF‐16, the effect of *tbc‐2* deletion on stress resistance in *daf‐2;daf‐16* deletion mutants was examined. The loss of *tbc‐2* significantly decreased resistance to chronic oxidative stress in *daf‐2;daf‐16* worms. A minimum of three biological replicates were performed. Statistical significance was assessed using the log‐rank test for panels (a), (b), (c), and (h). The overall *p*‐value for these panels is indicated. *p*‐values for comparisons between pairs of survival plots can be found in Table S1. Statistical significance was assessed using a two‐way ANOVA with Šidák's multiple comparison test for panels (d), (e), (f), and (g). **p < 0.01

Long‐lived insulin/IGF‐1 receptor mutants, *daf‐2*, have increased resistance to multiple external stressors, which is dependent on DAF‐16 (Dues et al., 2019). We previously showed that deletion of *tbc‐2* disrupts DAF‐16 localization in *daf‐2* mutants and decreases their lifespan (Meraş et al., 2022). Accordingly, we wondered whether disruption of *tbc‐2* would affect resistance to stress in *daf‐2* mutants. As in wild‐type worms, deletion of *tbc‐2* decreased resistance to heat stress in *daf‐2* worms (Figure 4a). Disruption of *tbc‐2* also decreased resistance to chronic oxidative stress in *daf‐2* mutants (Figure 4b) but had no effect on resistance to acute oxidative stress (Figure 4c). The loss of *tbc‐2* reduced *daf‐2* worms' resistance to osmotic stress (Figure 4d,e), anoxia (Figures 4f,g and S4), and bacterial pathogens (Figure 4h).

### 2.4 The effect of TBC‐2 disruption on resistance to stress is largely abolished in DAF‐16 mutant background

Since TBC‐2 affects the nuclear localization of DAF‐16, it is plausible that the effects of *tbc‐2* deletion on stress resistance are mediated by DAF‐16. To determine whether *tbc‐2* can alter stress resistance independently of DAF‐16, we examined the effect of disrupting *tbc‐2* on stress resistance of *daf‐16* mutants and *daf‐2;daf‐16* double mutants. The loss of *tbc‐2* in *daf‐16* mutants did not significantly decrease resistance to heat stress (Figure 3a), chronic oxidative stress (Figure 3b), acute oxidative stress (Figure 3c), osmotic stress (Figure 3d,e), anoxia (Figure 3f,g), or bacterial pathogen resistance (Figure 3h).

The loss of *tbc‐2* in *daf‐2;daf‐16* mutants did not significantly affect resistance to heat stress (Figure 4a) and but did decrease resistance to chronic oxidative stress (Figure 4b). In contrast, disruption of *tbc‐2* increased resistance to acute oxidative stress in *daf‐2;daf‐16* mutants (Figure 4c). Loss of *tbc‐2* did not significantly affect resistance to osmotic stress (Figure 4d,e), anoxia (Figure 4f,g) or bacterial pathogens (Figure 4h) in *daf‐2;daf‐16* mutants. Combined, these results indicate that the effect of disruption of *tbc‐2* on resistance to stress is lost in the absence of DAF‐16 for most stresses. This result is consistent with the effect of TBC‐2 disruption on stress resistance being primarily mediated by DAF‐16.

### 2.5 Loss of TBC‐2 decreases lifespan in DAF‐16 mutant background

**FIGURE 5:**
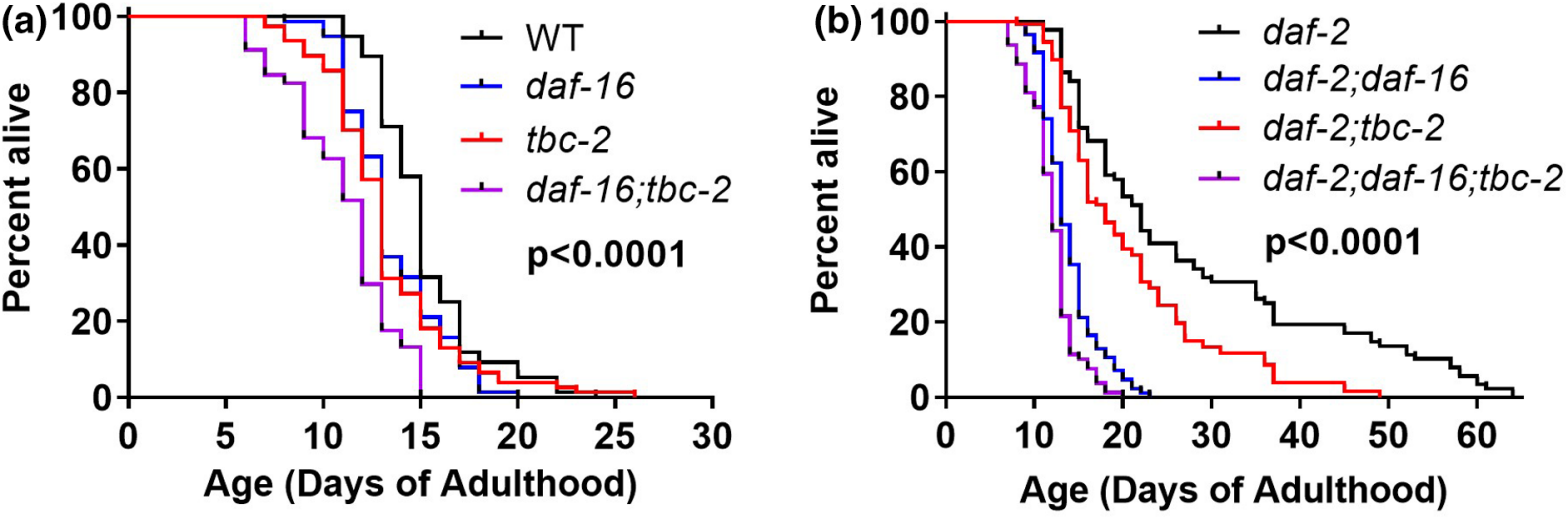
Disruption of TBC‐2 decreases lifespan in the presence and absence of DAF‐16. Disruption of *tbc‐2* decreases lifespan in wild‐type worms and *daf‐16* mutants (a). The loss of *tbc‐2* also shortens the lifespan of long‐lived *daf‐2* mutants and *daf‐2;daf‐16* double mutants (b). This indicates that deletion of *tbc‐2* can affect lifespan independently of DAF‐16. Three biological replicates were performed. Statistical significance was assessed using the log‐rank test. The overall *p*‐values are indicated. *p*‐values for comparisons between pairs of survival plots can be found in Table S1. Raw lifespan data can be found in Table S2.

As stress resistance is strongly correlated with longevity (Soo et al., 2022), we next examined the effect of disrupting *tbc‐2* on lifespan. In wild‐type worms, the loss of *tbc‐2* results in a small but significant decrease in lifespan (Figure 5a). As we have observed previously (Meraş et al., 2022), *tbc‐2* deletion markedly reduces the long lifespan of *daf‐2* mutants (Figure 5b). To determine whether the effects of *tbc‐2* on lifespan are mediated by DAF‐16, we examined the effect of disruption of *tbc‐2* in *daf‐16* mutant backgrounds. In both *daf‐16* and *daf‐2;daf‐16* worms, loss of *tbc‐2* resulted in a significant decrease in lifespan, similar to what was observed in wild‐type worms (Figure 5a,b). This indicates that at least some of the effects of *tbc‐2* on lifespan are independent of DAF‐16.

### 2.6 TBC‐2 is required for full nuclear localization of DAF‐16 in *daf‐2* mutants

**FIGURE 6:**
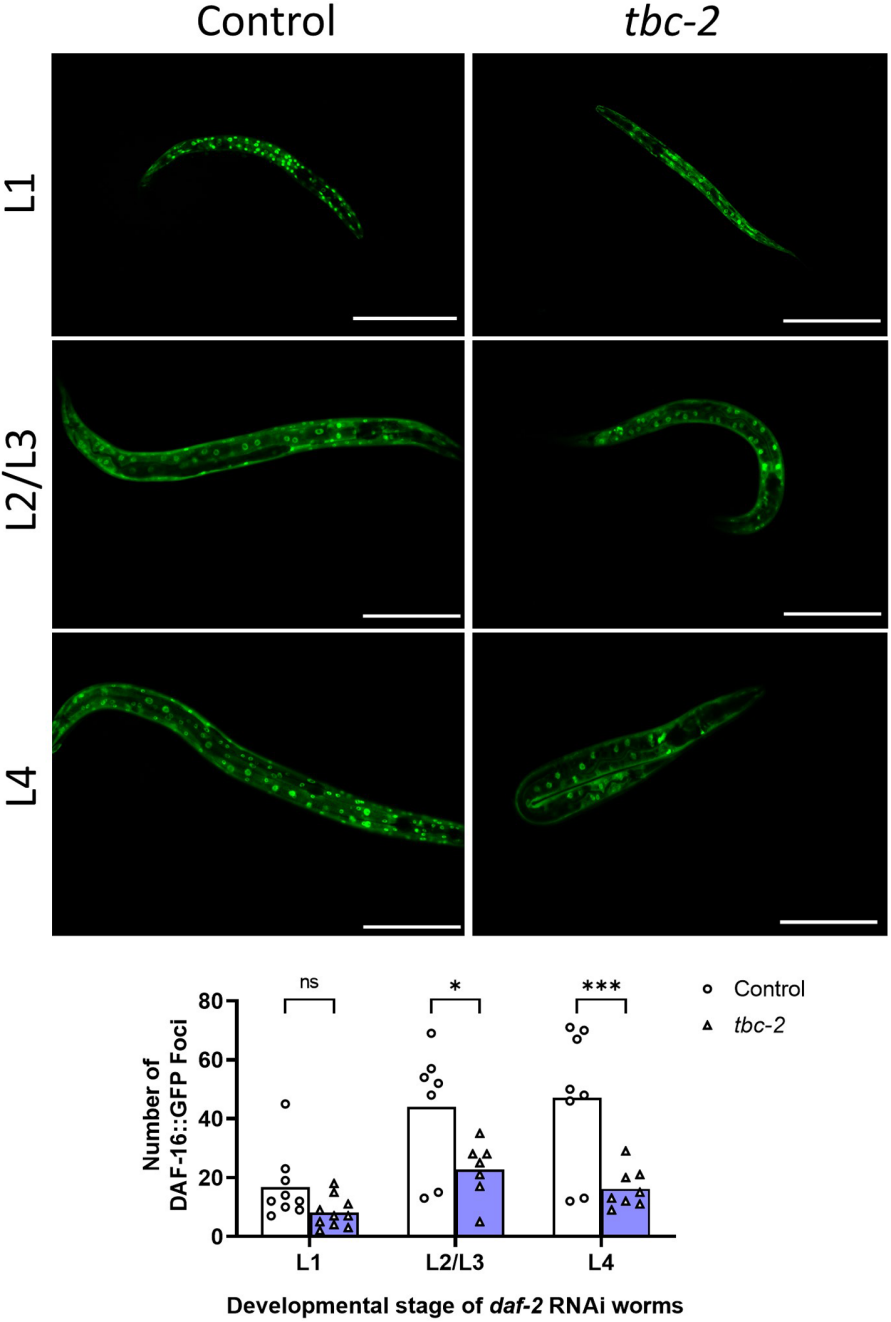
Loss of *tbc‐2* decreases nuclear localization of DAF‐16 following knockdown of *daf‐2* using RNAi. Knockdown of *daf‐2* expression was begun at the egg stage using RNAi by feeding in a wild‐type and *tbc‐2* background. Nuclear localization of DAF‐16 was monitored using a translational fusion strain: *zIs356*[*daf‐16p*::*daf‐16::GFP*]. While the knockdown of *daf‐2* resulted in nuclear localization of DAF‐16 in both backgrounds, inhibition of *tbc‐2* resulted in a significant decrease in nuclear localization. Scale bars indicate 100 μm. *N* = 7–10 animals per group. Statistical significance was assessed using mixed‐effects analysis and Šidák's multiple comparisons test. *p < 0.05, ***p < 0.001

As disruption of *tbc‐2* decreases stress resistance and lifespan in *daf‐2* mutants, we next sought to determine the role of TBC‐2 in the nuclear localization of DAF‐16 in *daf‐2* worms. For this purpose, we used RNAi to decrease *daf‐2* expression beginning at the egg stage. We also examined the effect of a *tbc‐2* deletion on the nuclear localization of DAF‐16 in *daf‐2* mutants. We found that *daf‐2* RNAi resulted in the nuclear localization of DAF‐16 in both wild‐type and *tbc‐2* mutant backgrounds. However, at each time point examined after *daf‐2* RNAi, there was significantly less nuclear localization of DAF‐16 in *tbc‐2* mutants compared with controls (Figure 6). Similarly, *daf‐2;tbc‐2* mutants have significantly decreased nuclear localization of DAF‐16 compared with *daf‐2* mutants (Figure S5). Combined, this indicates that TBC‐2 is needed for full nuclear localization of DAF‐16 in *daf‐2* mutants.

### 2.7 Intestinal TBC‐2 partially rescues deficits in lifespan and resistance to stress

To examine the extent to which loss of TBC‐2 in the intestine is required for the effect of TBC‐2 on stress resistance and lifespan, we expressed wild‐type TBC‐2 linked to GFP in the intestine of *daf‐2;tbc‐2* mutants (*daf‐2;tbc‐2;vhIs12[vha‐6p::GFP::tbc‐2]* worms). We previously showed that intestinal expression of GFP::TBC‐2 can rescue the enlarged endosome phenotype in *tbc‐2* mutants (Chotard et al., 2010). We found that expression of GFP::TBC‐2 in the intestine of *daf‐2;tbc‐2* worms significantly increased resistance to both chronic oxidative stress (4 mM paraquat; Figure S6a) and bacterial pathogens (*P. aeruginosa* strain PA14; Figure S6b). *daf‐2;tbc‐2;vhIs12[vha‐6p::GFP::tbc‐2]* worms also exhibited a trend toward increased lifespan compared with *daf‐2;tbc‐2* mutants, which failed to reach significance (Figure S6c). Overall, these results are consistent with TBC‐2 acting in the intestine to affect stress resistance and lifespan. However, it is also possible that other tissues are involved as the rescue was incomplete.

## 3 DISCUSSION

The pathways that modulate DAF‐16 activation have been well studied (Sun et al., 2017; Yen et al., 2011). While most of these studies have focused on cytoplasmic versus nuclear localization of DAF‐16, our recent work demonstrates that DAF‐16 can be present at different sites within the cytoplasm. Specifically, DAF‐16 can be localized to endosomes (Meraş et al., 2022). Combined with previous work demonstrating endosomal localization of other components of the IIS pathway (Balbis et al., 2000; Bergeron et al., 2016; Braccini et al., 2015; Christoforidis et al., 1999; Khan et al., 1986; Marat et al., 2017; Naguib et al., 2015), this suggests that key steps of the IIS pathway may occur on endosomes. The importance of endosomes in IIS is supported by the fact that IIS increases the localization of DAF‐16 to endosomes, while disruption of IIS decreases endosomal localization of DAF‐16 (Meraş et al., 2022). Endosomes may also serve as a storage site for cytoplasmic DAF‐16 where it can be released to nucleus or may be involved in targeting DAF‐16 to the lysosome for degradation. Sequestering or degrading DAF‐16 would both be predicted to reduce the ability of DAF‐16 to translocate to the nucleus and upregulate its target genes.

### 3.1 TBC‐2 affects nuclear localization of DAF‐16 in response to stress

We previously showed that *tbc‐2* mutants exhibit an accumulation of enlarged late endosomes (Chotard et al., 2010) and a marked increase in the number of endosomes containing DAF‐16 (Meraş et al., 2022). As the amount of total DAF‐16 appears to be similar between wild‐type worms and *tbc‐2* mutants based on DAF‐16::GFP expression in the translational reporter strain *daf‐16p::daf‐16::GFP*, the increase in endosomal DAF‐16 likely results in decreased free cytoplasmic DAF‐16. Here, we show that disruption of *tbc‐2* results in decreased or delayed nuclear localization of DAF‐16 in response to multiple stresses, including heat stress, anoxia, and exposure to bacterial pathogens. The decreased nuclear localization of DAF‐16 in *tbc‐2* mutants may result from a decrease in free cytoplasmic DAF‐16 and or from endosomal DAF‐16 being slower or unable to translocate to the nucleus in response to these stresses. Interestingly, loss of *tbc‐2* has the opposite effect of increasing nuclear localization of DAF‐16 in response to chronic oxidative stress and osmotic stress.

Despite the differences in the effect of *tbc‐2* disruption on DAF‐16 nuclear localization in response to different stresses, we observed a diminished upregulation of DAF‐16 target genes in *tbc‐2* mutants in response to all stresses examined. For stresses in which *tbc‐2* disruption resulted in increased or unchanged nuclear localization of DAF‐16, the decreased activation of DAF‐16 target genes may be due to an effect of TBC‐2 on DAF‐16 activity that is independent of DAF‐16 nuclear localization. Alternatively, the diminished upregulation of DAF‐16 target genes could result from the effects of *tbc‐2* deletion on DAF‐16‐independent pathways that may also influence the expression of the DAF‐16 target genes. In fact, we have recently observed that there is a large degree of overlap in genes upregulated by different stress response pathways (Soo et al., 2022).

### 3.2 TBC‐2 has an important role in surviving exogenous stressors

Disruption of *daf‐16* results in decreased resistance to multiple stresses (Lin et al., 2018). Consistent with their decreased nuclear localization of DAF‐16 and reduced expression of DAF‐16 target genes in response to stress, *tbc‐2* mutants exhibit decreased survival when exposed to exogenous stressors compared with wild‐type worms. *tbc‐2* mutants have decreased nuclear localization of DAF‐16 in response to heat stress, anoxia, and bacterial pathogens and show significantly decreased resistance to these same three stresses (Table S3), suggesting that decreased nuclear localization of DAF‐16 contributes to the decrease in resistance to these stresses. *tbc‐2* mutants also have decreased resistance to acute oxidative stress. Although there was a trend toward decreased nuclear localization of DAF‐16 for acute oxidative stress at the 2‐ and 3‐h time points, they failed to reach significance. It is possible that the decreased resistance to acute oxidative stress in *tbc‐2* mutants may result from mechanisms that are at least partially independent of DAF‐16. We did not observe enhanced resistance to stresses in which *tbc‐2* deletion resulted in increased nuclear localization of DAF‐16, but these stresses also did not result in the upregulation of DAF‐16 target genes in *tbc‐2* mutants.

Long‐lived *daf‐2* mutant worms exhibit markedly increased resistance to multiple stress, which is highly dependent on DAF‐16 (Dues et al., 2019). As a result, these mutants provide a larger window to observe effects of *tbc‐2* on stress resistance. The disruption of *tbc‐2* in *daf‐2* worms decreased resistance to heat stress, chronic oxidative stress, osmotic stress, anoxia, and bacterial pathogen stress (Table S3). Similarly, disruption of *daf‐16* in *daf‐2* worms results in decreased resistance to these same stresses (Figure S7; Dues et al., 2019). In contrast to *daf‐2;daf‐16* worms that do not have any enhancement of stress resistance compared with wild‐type worms, *daf‐2;tbc‐2* mutants still exhibit increased resistance to heat stress, oxidative stress, anoxia, and bacterial pathogen stress compared with wild‐type worms (Figure S7). This is consistent with our observation that nuclear localization of DAF‐16 in response to stress is decreased by the loss of *tbc‐2* but not eliminated. Our data are consistent with the DAF‐16‐mediated stress response being present in *tbc‐2* mutants but the magnitude of the response being diminished.

### 3.3 DAF‐16‐dependent and independent effects of *tbc‐2* disruption on stress resistance and lifespan

In order to determine the DAF‐16 dependency of the effect of *tbc‐2* deletion on stress resistance and lifespan, we examined the effect of disrupting *tbc‐2* in wild‐type and *daf‐2* worms lacking DAF‐16. We reasoned that if *tbc‐2* is affecting a phenotype entirely through DAF‐16‐dependent mechanisms, then (1) the *tbc‐2* mutation should not worsen the phenotype of *daf‐16* or *daf‐2;daf‐16* mutants; and (2) the phenotype of *daf‐16* and *daf‐2;daf‐16* mutants should be more severe or equivalent to *tbc‐2* and *daf‐2;tbc‐2* mutants, respectively. If *tbc‐2* is affecting a phenotype through DAF‐16‐independent mechanisms, then (1) *daf‐16;tbc‐2* and *daf‐2;daf‐16;tbc‐2* mutants should have a more severe phenotype than *daf‐16* mutants and *daf‐2;daf‐16* mutants, respectively; and (2) the phenotype of *daf‐16* and *daf‐2;daf‐16* mutants could be worse, better, or the same compared with *tbc‐2* and *daf‐2;tbc‐2* mutants, respectively. By these criteria, the effect of *tbc‐2* deletion on resistance to stress appears to be primarily DAF‐16‐dependent, while DAF‐16‐independent mechanisms appear to contribute to the effect of *tbc‐2* deletion on lifespan (Table S4).

As accumulating evidence suggests a role of endosomal trafficking in signal transduction (Bergeron et al., 2016; Miaczynska, 2013), the DAF‐16‐independent effects of *tbc‐2* deletion on stress resistance and lifespan may result from the disruption of other stress signaling pathways, which are dependent on endosomal trafficking through a similar mechanism to DAF‐16. There are a number of different signaling pathways that are associated with endosomes (Murphy et al., 2009) and multiple of these pathways have been shown to affect lifespan. For example, the nuclear hormone receptor NHR‐49 can be sequestered to endosomes (Watterson et al., 2022) and decreasing levels of NHR‐49 has been shown to decrease longevity (Khan et al., 2013; Walter et al., 2011). Similarly, epidermal growth factor (EGF) signaling has been shown to take place on endosomes (Wang et al., 2002). Gain‐of‐function mutants in the *C. elegans* EGF receptor homolog *let‐23* have increased lifespan (Yu & Driscoll, 2011), while disruption of the EGF ligand gene *lin‐3* decreases lifespan (Iwasa et al., 2010). Notch signaling is also associated with endosomes (Fortini & Bilder, 2009; Zheng et al., 2013). Disruption of the notch receptor gene *glp‐1* increases lifespan (Arantes‐Oliveira et al., 2002). Similarly, RNAi against *sel‐5*, which is a positive regulator of notch signaling, increases lifespan (Curran & Ruvkun, 2007), while disruption of *osm‐11*, which promotes notch signaling, also increases lifespan (Dresen et al., 2015). In future studies, it will be important to determine the extent to which other stress signaling pathways contribute to the decreased stress resistance and lifespan caused by disruption of *tbc‐2*.

### 3.4 Model for the effect of TBC‐2 on stress resistance and lifespan

**FIGURE 7:**
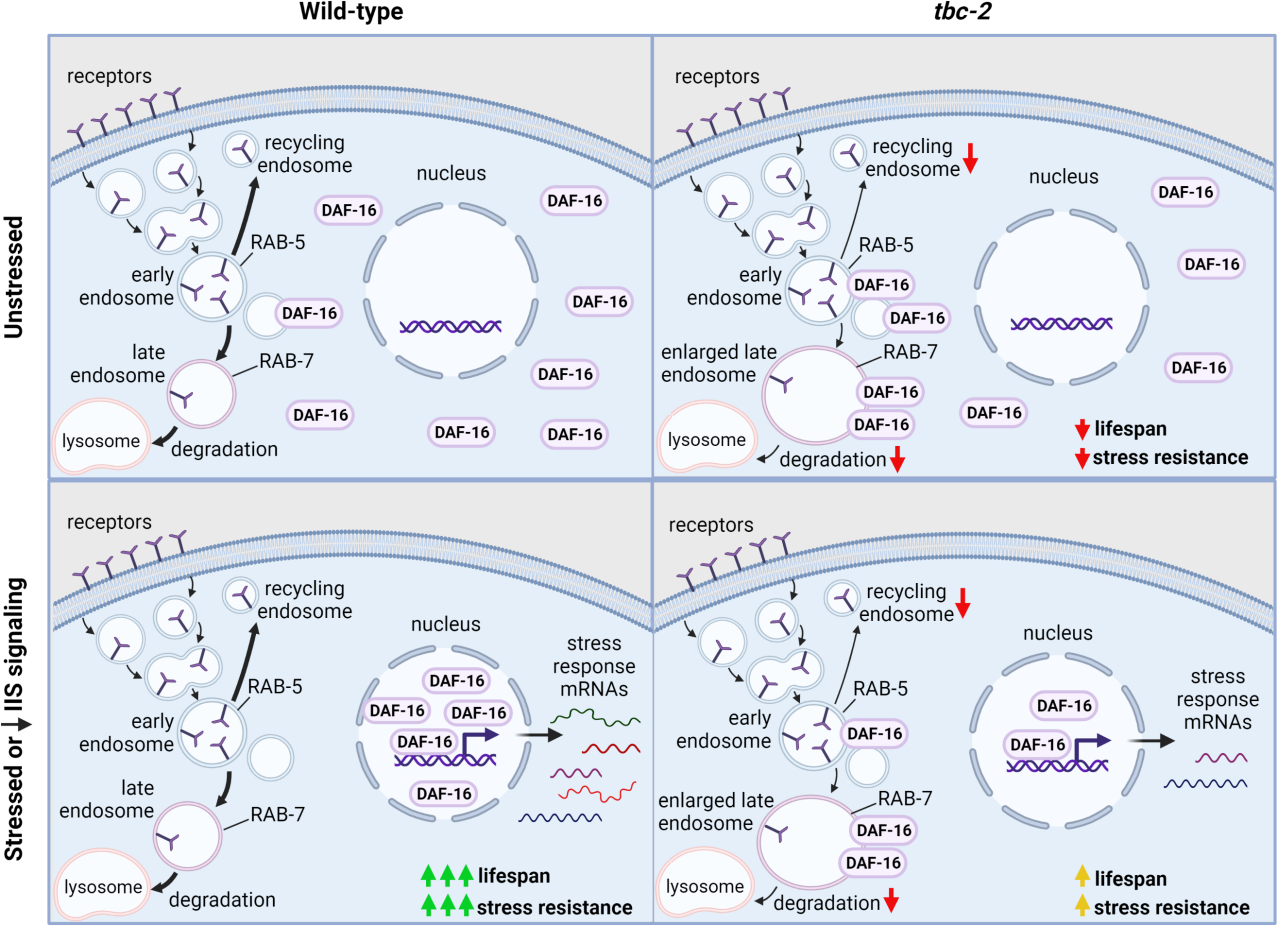
Model for the role of TBC‐2 in stress resistance and lifespan. In wild‐type worms under unstressed conditions, a small amount of cytoplasmic DAF‐16 is bound to endosomes while the majority of DAF‐16 is free in the cytoplasm. When IIS is decreased or under conditions of stress, cytoplasmic DAF‐16 translocates to the nucleus, thereby decreasing the amount of free cytoplasmic and endosomal DAF‐16. In the nucleus, DAF‐16 increases the expression of stress response genes, which results in increased stress resistance and lifespan. Under unstressed conditions, when *tbc‐2* is disrupted, there is an accumulation of enlarged late endosomes resulting from increased activity of RAB‐5. *tbc‐2* mutants exhibit an increase in endosomal DAF‐16, which likely results in a corresponding decrease in free cytoplasmic DAF‐16. When IIS is decreased in *tbc‐2* mutants or these mutants are exposed to stress, the translocation of DAF‐16 to the nucleus is decreased or delayed. This results in a decreased upregulation of stress response genes, which contributes to decreased stress resistance and lifespan in *tbc‐2* mutants. There may be similar mechanisms involved for other stress‐responsive transcription factors, thereby resulting in the DAF‐16‐independent effects of *tbc‐2* disruption on stress resistance and lifespan.

Based on the results of this work as well as our previous research on TBC‐2, we propose a model for the effect of *tbc‐2* on stress resistance and lifespan (Figure 7). In wild‐type worms that are unstressed, a small amount of DAF‐16 exists on endosomes while the majority exists as free cytoplasmic DAF‐16 (Meraş et al., 2022). In contrast, *tbc‐2* mutants have a marked increase in endosomal DAF‐16 (Meraş et al., 2022). The disruption of *tbc‐2* releases inhibition of RAB‐5 and RAB‐7 leading to the accumulation of enlarged endosomes (Chotard et al., 2010). This phenotype results from the increased activity of RAB‐5, as the enlarged endosome phenotype is also observed in constitutively active *rab‐5* mutants (Chotard et al., 2010). Although the reduction in RAB‐7 inhibition would be predicted to increase endosomal degradation at the lysosome, it is likely that when both RAB‐5 and RAB‐7 are uninhibited that the degradation at the lysosome fails to keep up with the increased rate of endosomal maturation mediated by RAB‐5. This imbalance may also result from the fact that TBC‐2 has greater GAP activity toward RAB‐5 than RAB‐7 (Chotard et al., 2010).

Under stressed conditions or when IIS is decreased, the free cytoplasmic DAF‐16 in wild‐type worms rapidly translocates to the nucleus to upregulate genes involved in stress resistance and metabolism. Short‐term activation of DAF‐16 facilitates the survival of acute stresses, while long‐term activation increases both stress resistance and lifespan. In contrast, *tbc‐2* mutants have altered nuclear localization of DAF‐16 (Figure 1) and decreased upregulation of DAF‐16 target genes in response to stress (Figure 2). As a result, disruption of *tbc‐2* decreases stress resistance (Figures 3 and 4) and lifespan (Figure 6) in wild‐type worms and *daf‐2* mutants. Finally, the ability of *tbc‐2* disruption to decrease lifespan in the absence of DAF‐16 (Figures 5, S7 and S8) likely results from effects of *tbc‐2* deletion on other stress signaling pathways that are dependent on endosomal trafficking through similar mechanisms to DAF‐16.

In the future, it will be important to determine the exact mechanisms by which DAF‐16 interacts with endosomes and whether this interaction is direct or indirect. Once this has been determined, disrupting or enhancing the interaction between DAF‐16 and endosomes and examining stress resistance and lifespan will provide further understanding of how endosomal localization of DAF‐16 affects DAF‐16 nuclear localization, DAF‐16 target gene expression, stress resistance, and lifespan.

## 4 CONCLUSIONS

In this work, we show that disruption of endosomal trafficking can decrease stress resistance and lifespan through mislocalization of stress‐responsive transcription factors. Specifically, deletion of *tbc‐2* results in increased localization of DAF‐16 to endosomes, which alters its ability to move to the nucleus in response to stress. *tbc‐2* mutants have decreased resistance to multiple exogenous stressors and decreased lifespan resulting from both DAF‐16‐dependent and DAF‐16‐independent mechanisms. Overall, this work demonstrates the importance of endosomal trafficking for stress resistance and lifespan by ensuring proper localization of stress‐responsive transcription factors.

## 5 EXPERIMENTAL PROCEDURES

### 5.1 Strains

N2 wild‐type

QR15 *tbc‐2(tm2241) II*

TJ356 *zIs356[daf‐16p::daf‐16::GFP] IV*

QR807 *tbc‐2(tm2241) II; zIs356[daf‐16p::daf‐16::GFP] IV*

CF1038 *daf‐16(mu86) I*

CB1370 *daf‐2(e1370) III*

JVR327 *daf‐16(mu86) I; daf‐2(e1370) III*

JVR596 *daf‐16(mu86) I; tbc‐2(tm2241) II*

QR150 *tbc‐2(tm2241) II; daf‐2(e1370) III*

JVR597 *daf‐16(mu86) I; tbc‐2(tm2241) II; daf‐2(e1370) III*

JVR304 *daf‐2(e1370) III; zIs356[daf‐16p::daf‐16::GFP] IV*

QR328 *tbc‐2(tm2241) II; daf‐2(e1370) III*; *zIs356[daf‐16p::daf‐16::GFP] IV*

QR870 *tbc‐2(tm2241) II; daf‐2(e1370) III; vhIs12[vha‐6p::GFP::TBC‐2]*

### 5.2 Nuclear localization of DAF‐16

DAF‐16 localization to the nucleus was imaged in young adult hermaphrodite worms at the level of the whole body using the DAF‐16::GFP (zIs356) reporter in either a wild‐type or *tbc‐2* background. Worms were incubated at 37°C for 0.5, 1, 2, and 4 h for exposure to heat stress; transferred to plates containing 4 mM paraquat with 100 μM FUdR for 24, 48 and 72 h for exposure to chronic oxidative stress; transferred to plates containing 220 mM paraquat with 100 μM FUdR for 1, 3, and 6 h for exposure to acute oxidative stress; transferred to plates containing 300 μM juglone for 1, 2, and 3 h for exposure to acute oxidative stress; transferred to 400 mM NaCl plates for 24 h for exposure to osmotic stress; put in BD Bio‐Bag Type A Environmental Chambers (Becton, Dickinson and Company) for 18, 24, and 48 h for exposure to anoxic stress; and exposed to *Pseudomonas aeruginosa* strain PA14 for 24 h for exposure to bacterial pathogens. Immediately after exposure to each exogenous stress, worms were mounted onto 2% agarose pads and immobilized with 5–10 μl of 10 mM levamisole. Worms were then imaged using an Axio Imager A1 microscope with a 40× objective lens, and images were captured using an AxioCam MRm camera and AxioVision software. Images were analyzed using Fiji (ImageJ) where the number of GFP‐positive foci was determined using the particle analysis tool. Approximately 20 worms were imaged over three replicates, per stress condition.

For imaging of DAF‐16 localization in response to *daf‐2* RNAi, eggs were transferred to NGM plates containing 25 μg/ml Carbenicillin and 3 mM IPTG seeded with 5× concentrated bacteria expressing dsRNA against *daf‐2*. For imaging of DAF‐16 localization in response to *daf‐2* RNAi and for imaging of DAF‐16 localization in *daf‐2;tbc‐2* mutants, worms of different age groups were mounted on 2% agarose pads and immobilized with 5–10 μl of 10 mM levamisole. Worms were imaged using an LSM780 confocal microscope and analyzed using ImageJ. Approximately 10 animals were imaged per age group.

### 5.3 Expression of DAF‐16 target genes in response to stress

To determine the effect of *tbc‐2* disruption on the ability of worms to upregulate DAF‐16 target genes in response to stress, we exposed WT and *tbc‐2* worms to exogenous stressors and then isolated their mRNA, as we have done previously (Soo et al., 2021). For exposure to heat stress, worms were incubated at 35°C for 2 h and then allowed to recover at 20°C for 24 h. For exposure to oxidative stress, worms were grown on 4 mM paraquat with 100 μM FUdR for 48 h. For exposure to anoxic stress, worms were placed in a BD Bio‐Bag Type A Environmental Chambers (Becton, Dickinson and Company) for 48 h and then allowed to recover for 24 h. For exposure to osmotic stress, worms were transferred to 400 mM NaCl plates for 24 h. For bacterial pathogen stress, worms were grown on pathogenic *Pseudomonas aeruginosa* (PA14) bacteria for 24 h. The unstressed control for heat stress, anoxic stress, osmotic stress, and bacterial pathogen stress is worms that were transferred to new plates as young adults and allowed to grow in normal conditions for 24 h before collection for mRNA isolation. The unstressed control for oxidative stress is worms that were transferred to 100 μM FUdR plates as young adults and allowed to grow in normal conditions for 48 h before collection for mRNA isolation. Three biological replicates were collected per condition.

### 5.4 Quantification of mRNA levels by quantitative RT‐PCR

To perform quantitative RT‐PCR, we first collected worms in M9 buffer and extracted RNA using Trizol as described previously (Machiela et al., 2016). Using a High‐Capacity cDNA Reverse Transcription kit (Applied Biosystems 4368814), the isolated mRNA was then converted to cDNA. Quantitative PCR was performed using a PowerUp SYBR Green Master Mix (Applied Biosystems A25742) in a MicroAmp Optical 96‐Well Reaction Plate (Applied Biosystems N8010560) and a Viia 7 Applied Biosystems qPCR machine. mRNA levels were calculated as the copy number of the gene of interest relative to the copy number of the endogenous control, *act‐3*, and then expressed as a percentage of the unstressed control. Primer sequences for each target gene are as follows:

*sod‐3* (AAAGGAGCTGATGGACACTATTAAGC, AAGTTATCCAGGGAACCGAAGTC),

*dod‐3* (AAGTGCTCCGATTGTTACGC, ACATGAACACCGGCTCATTC),

*mtl‐1* (ATGGCTTGCAAGTGTGACTG, GCTTCTGCTCTGCACAATGA),

*sodh‐1* (GAAGGAGCTGGAAGTGTTGTTC, CTCCACGTATAGTGAGGTACTCCTG),

*ftn‐1* (GAGTGGGGAACTGTCCTTGA, CGAATGTACCTGCTCTTCCA),

*icl‐1* (TGTGAAGCCGAGGACTACCT, TCTCCGATCCAAGCTGATCT),

*act‐3* (TGCGACATTGATATCCGTAAGG, GGTGGTTCCTCCGGAAAGAA).

### 5.5 Heat stress assay

To measure resistance to heat stress, approximately 25 pre‐fertile young adult worms were transferred to new NGM plates freshly seeded with OP50 bacteria and were incubated at 37°C. Survival was measured every 2 h for a total of 8 h of incubation.

### 5.6 Oxidative stress assays

Resistance to chronic oxidative stress was measured by transferring approximately 30 pre‐fertile young adult worms to plates containing 4 mM paraquat and 100 μM FUdR and seeded with OP50 bacteria. Worms were kept at 20°C, and survival was monitored daily until all worms died. Resistance to acute oxidative stress was measured by transferring approximately 25 pre‐fertile young adult worms to 300 μM juglone plates seeded with OP50 bacteria. Worms were kept at 20°C, and survival was monitored every 2 h for a total of 8 h. See (Senchuk et al., 2017) for detailed protocols of both oxidative stress assays.

### 5.7 Osmotic stress assay

To measure resistance to osmotic stress, approximately 25 pre‐fertile young adult worms were transferred to NGM plates containing 400 or 500 mM NaCl and seeded with OP50 bacteria. Worms were kept at 20°C for 24 h before survival was scored.

### 5.8 Anoxia assay

To measure resistance to anoxic stress, approximately 25 pre‐fertile young adult worms were transferred to new NGM plates seeded with OP50 bacteria. To create a low‐oxygen environment for the worms, we utilized Becton‐Dickinson Bio‐Bag Type A Environmental Chambers. Worms were left in the Bio‐Bags for 48–96 h at 20°C and then allowed to recover for 24 h at 20°C before survival was measured.

### 5.9 Bacterial pathogen stress assay

We employed the slow kill bacterial pathogen assay to test for nematode resistance to death by bacterial colonization of the intestine. The slow kill assay was performed as described previously (Campos et al., 2021; Wu et al., 2019). OP50 bacteria were seeded to the center of NGM plates containing 100 mg/L FUdR, and plates were left at room temperature for 2 days. PA14 cultures were grown with aeration at 37°C for 16 h and then seeded to the center of NGM agar plates containing 20 mg/L FUdR. The plates containing PA14 bacteria were incubated at 37°C for 24 h and then at room temperature for 24 h. Approximately 40 L4 worms were transferred to plates containing 100 mg/L FUdR that were seeded with OP50 bacteria, and the worms were grown at 20°C until they reached day 3 of adulthood. Day 3 adult worms were then transferred from these plates onto plates containing 20 mg/L FUdR that were seeded with PA14 bacteria. The assay was conducted at 20°C, and survival was monitored daily until all worms died.

### 5.10 Lifespan assay

Lifespan assays were completed at 20°C and on NGM agar plates that contained FUdR to inhibit the development of progeny and limit internal hatching. We used a low concentration of 25 μM FUdR, which we have previously shown does not affect the longevity of wild‐type worms (Van Raamsdonk & Hekimi, 2011). For each lifespan assay, 40 pre‐fertile young adult worms were transferred to 25 μM FUdR plates seeded with OP50 bacteria and were kept at 20°C. Three biological replicates were completed, with genotypes being blinded in each replicate. Replicate start days were staggered, and survival of the worms was checked every other day. Worms were excluded from the assay if they crawled off the agar and died on the side of the plate, had internal hatching of progeny or expulsion of internal organs.

### 5.11 Statistical analysis

Biological replicates and or N are indicated in the figure legends. In most cases, we completed three biological replicates. In each replicate, an individual population of worms was tested. Where possible, the experimenter was blinded to the genotype during the course of the experiment, to ensure unbiased results. Statistical significance of differences between groups was determined by computing a *t* test, a one‐way ANOVA with Dunnett's multiple comparison test, a two‐way ANOVA or mixed‐effects analysis with Šidák's multiple comparisons test, or a log‐rank test using GraphPad Prism, as indicated in the figure legends. All error bars indicate the standard error of the mean.

## AUTHOR CONTRIBUTIONS

CR and JVR involved in conceptualization. AT, SS, AA, BK, and JVR involved in methodology and investigation. AT, SS, AA, BK, and JVR involved in analysis and visualization. JVR involved in writing—original draft and supervision. AT, SS, AA, BK, CR, and JVR involved in writing—review and editing.

## FUNDING INFORMATION

This work was supported by the Canadian Institutes of Health Research (CIHR; http://www.cihr‐irsc.gc.ca/; JVR) and the Natural Sciences and Engineering Research Council of Canada (NSERC; https://www.nserc‐crsng.gc.ca/index_eng.asp; JVR). JVR received a salary award from Fonds de Recherche du Quebec Santé (FRQS) and Parkinson Quebec. CR is supported by a project grant from the CIHR (PJT‐159725). AT received scholarships from NSERC and FRQS. The funders had no role in study design, data collection and analysis, decision to publish, or preparation of the manuscript.

## CONFLICT OF INTEREST

The authors declare that no conflicts of interest exist.

## Supporting information


FigureS1‐S8

TableS1‐S4

TableS2

## Data Availability

Raw data for all experiments are contained within the manuscript or available upon request by contacting jeremy.vanraamsdonk@mcgill.ca.

## References

[acel13762-bib-0001] Apfeld, J. , & Kenyon, C. (1999). Regulation of lifespan by sensory perception in *Caenorhabditis elegans* . Nature, 402(6763), 804–809. 10.1038/45544 10617200

[acel13762-bib-0002] Arantes‐Oliveira, N. , Apfeld, J. , Dillin, A. , & Kenyon, C. (2002). Regulation of life‐span by germ‐line stem cells in *Caenorhabditis elegans* . Science, 295(5554), 502–505. 10.1126/science.1065768 11799246

[acel13762-bib-0003] Balbis, A. , Baquiran, G. , Bergeron, J. J. , & Posner, B. I. (2000). Compartmentalization and insulin‐induced translocations of insulin receptor substrates, phosphatidylinositol 3‐kinase, and protein kinase B in rat liver. Endocrinology, 141(11), 4041–4049. 10.1210/endo.141.11.7774 11089534

[acel13762-bib-0004] Barsyte, D. , Lovejoy, D. , & Lithgow, G. (2001). Longevity and heavy metal resistance in daf‐2 and age‐1 long‐lived mutants of *Caenorhabditis elegans* . The FASEB Journal, 15(3), 627–634. 10.1096/fj.99-0966com 11259381

[acel13762-bib-0005] Bergeron, J. J. , Di Guglielmo, G. M. , Dahan, S. , Dominguez, M. , & Posner, B. I. (2016). Spatial and temporal regulation of receptor tyrosine kinase activation and intracellular signal transduction. Annual Review of Biochemistry, 85, 573–597. 10.1146/annurev-biochem-060815-014659 27023845

[acel13762-bib-0006] Berman, J. R. , & Kenyon, C. (2006). Germ‐cell loss extends *C. elegans* life span through regulation of DAF‐16 by kri‐1 and lipophilic‐hormone signaling. Cell, 124(5), 1055–1068. 10.1016/j.cell.2006.01.039 16530050

[acel13762-bib-0007] Braccini, L. , Ciraolo, E. , Campa, C. C. , Perino, A. , Longo, D. L. , Tibolla, G. , Prenolato, M. , Cao, Y. , Tassone, B. , Damilano, F. , Laffargue, M. , Calautti, E. , Falasca, M. , Norata, G. D. , Backer, J. M. , & Hirsch, E. (2015). PI3K‐C2gamma is a Rab5 effector selectively controlling endosomal Akt2 activation downstream of insulin signalling. Nature Communications, 6, 7400. 10.1038/ncomms8400 PMC4479417 26100075

[acel13762-bib-0008] Campos, J. C. , Wu, Z. , Rudich, P. D. , Soo, S. K. , Mistry, M. , Ferreira, J. C. , Blackwell, T. K. , & Van Raamsdonk, J. M. (2021). Mild mitochondrial impairment enhances innate immunity and longevity through ATFS‐1 and p38 signaling. EMBO Reports, 22(12), e52964. 10.15252/embr.202152964 34617666 PMC8647147

[acel13762-bib-0009] Chen, A. T.‐Y. , Guo, C. , Itani, O. A. , Budaitis, B. G. , Williams, T. W. , Hopkins, C. E. , McEachin, R. C. , Pande, M. , Grant, A. R. , Yoshina, S. , Mitani, S. , & Hu, P. J. (2015). Longevity genes revealed by integrative analysis of isoform‐specific daf‐16/FoxO mutants of *Caenorhabditis elegans* . Genetics, 201(2), 613–629. 26219299 10.1534/genetics.115.177998 PMC4596673

[acel13762-bib-0010] Chotard, L. , Mishra, A. K. , Sylvain, M. A. , Tuck, S. , Lambright, D. G. , & Rocheleau, C. E. (2010). TBC‐2 regulates RAB‐5/RAB‐7‐mediated endosomal trafficking in *Caenorhabditis elegans* . Molecular Biology of the Cell, 21(13), 2285–2296. 10.1091/mbc.E09-11-0947 20462958 PMC2893991

[acel13762-bib-0011] Christoforidis, S. , Miaczynska, M. , Ashman, K. , Wilm, M. , Zhao, L. , Yip, S. C. , Waterfield, M. D. , Backer, J. M. , & Zerial, M. (1999). Phosphatidylinositol‐3‐OH kinases are Rab5 effectors. Nature Cell Biology, 1(4), 249–252. 10.1038/12075 10559924

[acel13762-bib-0012] Curran, S. P. , & Ruvkun, G. (2007). Lifespan regulation by evolutionarily conserved genes essential for viability. PLoS Genetics, 3(4), e56. 10.1371/journal.pgen.0030056 17411345 PMC1847696

[acel13762-bib-0013] Dresen, A. , Finkbeiner, S. , Dottermusch, M. , Beume, J. S. , Li, Y. , Walz, G. , & Neumann‐Haefelin, E. (2015). *C. elegans* OSM‐11 signaling regulates SKN‐1/Nrf during embryonic development and adult longevity and stress response. Developmental Biology, 400, 118–131. 10.1016/j.ydbio.2015.01.021 25637691

[acel13762-bib-0014] Dues, D. J. , Andrews, E. K. , Schaar, C. E. , Bergsma, A. L. , Senchuk, M. M. , & Van Raamsdonk, J. M. (2016). Aging causes decreased resistance to multiple stresses and a failure to activate specific stress response pathways. Aging, 8(4), 777–795. 10.18632/aging.100939 27053445 PMC4925828

[acel13762-bib-0015] Dues, D. J. , Andrews, E. K. , Senchuk, M. M. , & Van Raamsdonk, J. M. (2019). Resistance to stress can Be experimentally dissociated from longevity. The Jurnals of Gerontology. Series A, Biological Sciences and Medical Sciences, 74(8), 1206–1214. 10.1093/gerona/gly213 PMC6625593 30247515

[acel13762-bib-0016] Fortini, M. E. , & Bilder, D. (2009). Endocytic regulation of notch signaling. Current Opinion in Genetics & Development, 19(4), 323–328. 10.1016/j.gde.2009.04.005 19447603 PMC2731830

[acel13762-bib-0017] Friedman, D. B. , & Johnson, T. E. (1988). A mutation in the age‐1 gene in *Caenorhabditis elegans* lengthens life and reduces hermaphrodite fertility. Genetics, 118(1), 75–86. 8608934 10.1093/genetics/118.1.75 PMC1203268

[acel13762-bib-0018] Garsin, D. A. , Villanueva, J. M. , Begun, J. , Kim, D. H. , Sifri, C. D. , Calderwood, S. B. , Ruvkun, G. , & Ausubel, F. M. (2003). Long‐lived *C. elegans* daf‐2 mutants are resistant to bacterial pathogens. Science, 300(5627), 1921. 10.1126/science.1080147 12817143

[acel13762-bib-0019] Hansen, M. , Taubert, S. , Crawford, D. , Libina, N. , Lee, S. J. , & Kenyon, C. (2007). Lifespan extension by conditions that inhibit translation in *Caenorhabditis elegans* . Aging Cell, 6(1), 95–110. 10.1111/j.1474-9726.2006.00267.x 17266679

[acel13762-bib-0020] Henderson, S. T. , & Johnson, T. E. (2001). Daf‐16 integrates developmental and environmental inputs to mediate aging in the nematode *Caenorhabditis elegans* . Current Biology, 11(24), 1975–1980. 10.1016/s0960-9822(01)00594-2 11747825

[acel13762-bib-0021] Honda, Y. , & Honda, S. (1999). The daf‐2 gene network for longevity regulates oxidative stress resistance and Mn‐superoxide dismutase gene expression in *Caenorhabditis elegans* . The FASEB Journal, 13(11), 1385–1393. 10.1096/fasebj.13.11.1385 10428762

[acel13762-bib-0022] Iwasa, H. , Yu, S. , Xue, J. , & Driscoll, M. (2010). Novel EGF pathway regulators modulate *C. elegans* healthspan and lifespan via EGF receptor, PLC‐gamma, and IP3R activation. Aging Cell, 9(4), 490–505. 10.1111/j.1474-9726.2010.00575.x 20497132 PMC5859306

[acel13762-bib-0023] Kenyon, C. (2005). The plasticity of aging: Insights from long‐lived mutants. Cell, 120(4), 449–460. 10.1016/j.cell.2005.02.002 15734678

[acel13762-bib-0024] Kenyon, C. , Chang, J. , Gensch, E. , Rudner, A. , & Tabtiang, R. (1993). A *C. elegans* mutant that lives twice as long as wild type. Nature, 366(6454), 461–464. 10.1038/366461a0 8247153

[acel13762-bib-0025] Khan, M. H. , Ligon, M. , Hussey, L. R. , Hufnal, B. , Farber, R., II , Munkácsy, E. , Rodriguez, A. , Dillow, A. , Kahlig, E. , & Rea, S. L. (2013). TAF‐4 is required for the life extension of isp‐1, clk‐1 and tpk‐1 Mit mutants. Aging, 5, 741–758. 24107417 10.18632/aging.100604 PMC3838777

[acel13762-bib-0026] Khan, M. N. , Savoie, S. , Bergeron, J. J. , & Posner, B. I. (1986). Characterization of rat liver endosomal fractions. In vivo activation of insulin‐stimulable receptor kinase in these structures. The Journal of Biological Chemistry, 261(18), 8462–8472. 3522569

[acel13762-bib-0027] Lakowski, B. , & Hekimi, S. (1998). The genetics of caloric restriction in *Caenorhabditis elegans* . Proceedings of the National Academy of Sciences of the United States of America, 95(22), 13091–13096. 10.1073/pnas.95.22.13091 9789046 PMC23719

[acel13762-bib-0028] Law, F. , & Rocheleau, C. E. (2017). Vps34 and the Armus/TBC‐2 Rab GAPs: Putting the brakes on the endosomal Rab5 and Rab7 GTPases. Cellular Logistics, 7(4), e1403530. 10.1080/21592799.2017.1403530 29296513 PMC5739090

[acel13762-bib-0029] Lin, K. , Dorman, J. B. , Rodan, A. , & Kenyon, C. (1997). Daf‐16: An HNF‐3/forkhead family member that can function to double the life‐span of *Caenorhabditis elegans* . Science, 278(5341), 1319–1322. 9360933 10.1126/science.278.5341.1319

[acel13762-bib-0030] Lin, X. X. , Sen, I. , Janssens, G. E. , Zhou, X. , Fonslow, B. R. , Edgar, D. , Stroustrup, N. , Swoboda, P. , Yates, J. R., III , Ruvkun, G. , & Riedel, C. G. (2018). DAF‐16/FOXO and HLH‐30/TFEB function as combinatorial transcription factors to promote stress resistance and longevity. Nature Communications, 9(1), 4400. 10.1038/s41467-018-06624-0 PMC6199276 30353013

[acel13762-bib-0031] Lithgow, G. J. , White, T. M. , Melov, S. , & Johnson, T. E. (1995). Thermotolerance and extended life‐span conferred by single‐gene mutations and induced by thermal stress. Proceedings of the National Academy of Sciences of the United States of America, 92(16), 7540–7544. 10.1073/pnas.92.16.7540 7638227 PMC41375

[acel13762-bib-0032] Liu, O. , & Grant, B. D. (2015). Basolateral endocytic recycling requires RAB‐10 and AMPH‐1 mediated recruitment of RAB‐5 GAP TBC‐2 to endosomes. PLoS Genetics, 11(9), e1005514. 10.1371/journal.pgen.1005514 26393361 PMC4578947

[acel13762-bib-0033] Machiela, E. , Dues, D. J. , Senchuk, M. M. , & Van Raamsdonk, J. M. (2016). Oxidative stress is increased in *C. elegans* models of Huntington's disease but does not contribute to polyglutamine toxicity phenotypes. Neurobiology of Disease, 96, 1–11. 10.1016/j.nbd.2016.08.008 27544481

[acel13762-bib-0034] Marat, A. L. , Wallroth, A. , Lo, W. T. , Müller, R. , Norata, G. D. , Falasca, M. , Schultz, C. , & Haucke, V. (2017). mTORC1 activity repression by late endosomal phosphatidylinositol 3,4‐bisphosphate. Science, 356(6341), 968–972. 10.1126/science.aaf8310 28572395

[acel13762-bib-0035] Meraş, I. , Chotard, L. , Liontis, T. , Ratemi, Z. , Wiles, B. , Seo, J. H. , Van Raamsdonk, J. M. , & Rocheleau, C. E. (2022). The Rab GTPase activating protein TBC‐2 regulates endosomal localization of DAF‐16 FOXO and lifespan. PLoS Genetics, 18(8), e1010328. 10.1371/journal.pgen.1010328 35913999 PMC9371356

[acel13762-bib-0036] Miaczynska, M. (2013). Effects of membrane trafficking on signaling by receptor tyrosine kinases. Cold Spring Harbor Perspectives in Biology, 5(11), a009035. 10.1101/cshperspect.a009035 24186066 PMC3809584

[acel13762-bib-0037] Murphy, C. T. , McCarroll, S. A. , Bargmann, C. I. , Fraser, A. , Kamath, R. S. , Ahringer, J. , Li, H. , & Kenyon, C. (2003). Genes that act downstream of DAF‐16 to influence the lifespan of *Caenorhabditis elegans* . Nature, 424(6946), 277–283. 10.1038/nature01789 12845331

[acel13762-bib-0038] Murphy, J. E. , Padilla, B. E. , Hasdemir, B. , Cottrell, G. S. , & Bunnett, N. W. (2009). Endosomes: A legitimate platform for the signaling train. Proceedings of the National Academy of Sciences of the United States of America, 106(42), 17615–17622. 10.1073/pnas.0906541106 19822761 PMC2764915

[acel13762-bib-0039] Naguib, A. , Bencze, G. , Cho, H. , Zheng, W. , Tocilj, A. , Elkayam, E. , Faehnle, C. R. , Jaber, N. , Pratt, C. P. , Chen, M. , Zong, W. X. , Marks, M. S. , Joshua‐Tor, L. , Pappin, D. J. , & Trotman, L. C. (2015). PTEN functions by recruitment to cytoplasmic vesicles. Molecular Cell, 58(2), 255–268. 10.1016/j.molcel.2015.03.011 25866245 PMC4423730

[acel13762-bib-0040] Ogg, S. , Paradis, S. , Gottlieb, S. , Patterson, G. I. , Lee, L. , Tissenbaum, H. A. , & Ruvkun, G. (1997). The fork head transcription factor DAF‐16 transduces insulin‐like metabolic and longevity signals in *C. elegans* . Nature, 389(6654), 994–999. 10.1038/40194 9353126

[acel13762-bib-0041] Schaar, C. E. , Dues, D. J. , Spielbauer, K. K. , Machiela, E. , Cooper, J. F. , Senchuk, M. , Hekimi, S. , & Van Raamsdonk, J. M. (2015). Mitochondrial and cytoplasmic ROS have opposing effects on lifespan. PLoS Genetics, 11(2), e1004972. 10.1371/journal.pgen.1004972 25671321 PMC4335496

[acel13762-bib-0042] Schenck, A. , Goto‐Silva, L. , Collinet, C. , Rhinn, M. , Giner, A. , Habermann, B. , Brand, M. , & Zerial, M. (2008). The endosomal protein Appl1 mediates Akt substrate specificity and cell survival in vertebrate development. Cell, 133(3), 486–497. 10.1016/j.cell.2008.02.044 18455989

[acel13762-bib-0043] Senchuk, M. M. , Dues, D. J. , Schaar, C. E. , Johnson, B. K. , Madaj, Z. B. , Bowman, M. J. , Winn, M. E. , & Van Raamsdonk, J. M. (2018). Activation of DAF‐16/FOXO by reactive oxygen species contributes to longevity in long‐lived mitochondrial mutants in *Caenorhabditis elegans* . PLoS Genetics, 14(3), e1007268. 10.1371/journal.pgen.1007268 29522556 PMC5862515

[acel13762-bib-0044] Senchuk, M. M. , Dues, D. J. , & Van Raamsdonk, J. M. (2017). Measuring oxidative stress in *Caenorhabditis elegans*: Paraquat and Juglone sensitivity assays. Bio‐Protocol, 7(1), e2086. 10.21769/BioProtoc.2086 29276721 PMC5739066

[acel13762-bib-0045] Shinde, S. R. , & Maddika, S. (2016). PTEN modulates EGFR late endocytic trafficking and degradation by dephosphorylating Rab7. Nature Communications, 7, 10689. 10.1038/ncomms10689 PMC4754336 26869029

[acel13762-bib-0046] Soo, S. K. , Traa, A. , Rudich, P. D. , Mistry, M. , & Van Raamsdonk, J. M. (2021). Activation of mitochondrial unfolded protein response protects against multiple exogenous stressors. Life Science Alliance, 4(12), e202101182. 10.26508/lsa.202101182 34583931 PMC8500221

[acel13762-bib-0047] Soo, S. K. , Traa, A. , Rudich, Z. D. , Moldakozhayev, A. , Mistry, M. , & Van Raamsdonk, J. M. (2022). Genetic basis of enhanced stress resistance in long‐lived mutants highlights key role of innate immunity in determining longevity. Aging Cell, e13740. 10.1111/acel.13740. Online ahead of print. 36514863 PMC9924947

[acel13762-bib-0048] Sun, X. , Chen, W. D. , & Wang, Y. D. (2017). DAF‐16/FOXO transcription factor in aging and longevity. Frontiers in Pharmacology, 8, 548. 10.3389/fphar.2017.00548 28878670 PMC5572328

[acel13762-bib-0049] Syntichaki, P. , Troulinaki, K. , & Tavernarakis, N. (2007). eIF4E function in somatic cells modulates ageing in *Caenorhabditis elegans* . Nature, 445(7130), 922–926. 10.1038/nature05603 17277769

[acel13762-bib-0050] Tepper, R. G. , Ashraf, J. , Kaletsky, R. , Kleemann, G. , Murphy, C. T. , & Bussemaker, H. J. (2013). PQM‐1 complements DAF‐16 as a key transcriptional regulator of DAF‐2‐mediated development and longevity. Cell, 154(3), 676–690. 10.1016/j.cell.2013.07.006 23911329 PMC3763726

[acel13762-bib-0051] Van Raamsdonk, J. M. , & Hekimi, S. (2011). FUdR causes a twofold increase in the lifespan of the mitochondrial mutant gas‐1. Mechanisms of Ageing and Development, 132(10), 519–521. 10.1016/j.mad.2011.08.006 21893079 PMC4074524

[acel13762-bib-0052] Walter, L. , Baruah, A. , Chang, H. W. , Pace, H. M. , & Lee, S. S. (2011). The homeobox protein CEH‐23 mediates prolonged longevity in response to impaired mitochondrial electron transport chain in *C. elegans* . PLoS Biology, 9(6), e1001084. 10.1371/journal.pbio.1001084 21713031 PMC3119657

[acel13762-bib-0053] Walz, H. A. , Shi, X. , Chouinard, M. , Bue, C. A. , Navaroli, D. M. , Hayakawa, A. , Zhou, Q. L. , Nadler, J. , Leonard, D. M. , & Corvera, S. (2010). Isoform‐specific regulation of Akt signaling by the endosomal protein WDFY2. The Journal of Biological Chemistry, 285(19), 14101–14108. 10.1074/jbc.M110.110536 20189988 PMC2863185

[acel13762-bib-0054] Wang, Y. , Pennock, S. , Chen, X. , & Wang, Z. (2002). Endosomal signaling of epidermal growth factor receptor stimulates signal transduction pathways leading to cell survival. Molecular and Cellular Biology, 22(20), 7279–7290. 10.1128/MCB.22.20.7279-7290.2002 12242303 PMC139821

[acel13762-bib-0055] Watterson, A. , Tatge, L. , Wajahat, N. , Arneaud, S. L. B. , Solano Fonseca, R. , Beheshti, S. T. , Metang, P. , Mihelakis, M. , Zuurbier, K. R. , Corley, C. D. , Dehghan, I. , McDonald, J. G. , & Douglas, P. M. (2022). Intracellular lipid surveillance by small G protein geranylgeranylation. Nature, 605(7911), 736–740. 10.1038/s41586-022-04729-7 35585236 PMC9885440

[acel13762-bib-0056] Wu, Z. , Isik, M. , Moroz, N. , Steinbaugh, M. J. , Zhang, P. , & Blackwell, T. K. (2019). Dietary restriction extends lifespan through metabolic regulation of innate immunity. Cell Metabolism, 29(5), 1192–1205 e1198. 10.1016/j.cmet.2019.02.013 30905669 PMC6506407

[acel13762-bib-0057] Yen, K. , Narasimhan, S. D. , & Tissenbaum, H. A. (2011). DAF‐16/Forkhead box O transcription factor: Many paths to a single fork(head) in the road. Antioxidants & Redox Signaling, 14(4), 623–634. 10.1089/ars.2010.3490 20673162 PMC3021330

[acel13762-bib-0058] Yu, S. , & Driscoll, M. (2011). EGF signaling comes of age: Promotion of healthy aging in *C. elegans* . Experimental Gerontology, 46(2–3), 129–134. 10.1016/j.exger.2010.10.010 21074601 PMC4841623

[acel13762-bib-0059] Zheng, L. , Saunders, C. A. , Sorensen, E. B. , Waxmonsky, N. C. , & Conner, S. D. (2013). Notch signaling from the endosome requires a conserved dileucine motif. Molecular Biology of the Cell, 24(3), 297–307. 10.1091/mbc.E12-02-0081 23171551 PMC3564540

