# Supplementary material for: Endosomal trafficking protein TBC‐2 modulates stress resistance and lifespan through DAF‐16‐dependent and independent mechanisms
Source: Aging Cell. 2023 Feb 15;22(3):e13762. doi: 10.1111/acel.13762 (PMC10014066; doi:10.1111/acel.13762)
Supplement: Supplementary file 1 — FigureS1‐S8 [file ACEL-22-e13762-s003.pdf]

Supporting Information for:

Endosomal trafficking protein TBC-2 modulates stress resistance and lifespan through DAF-16-dependent and independent mechanisms

Annika Traa, Sonja K. Soo, Abdelrahman AlOkda, Bokang Ko, Christian E. Rocheleau, Jeremy M. Van Raamsdonk

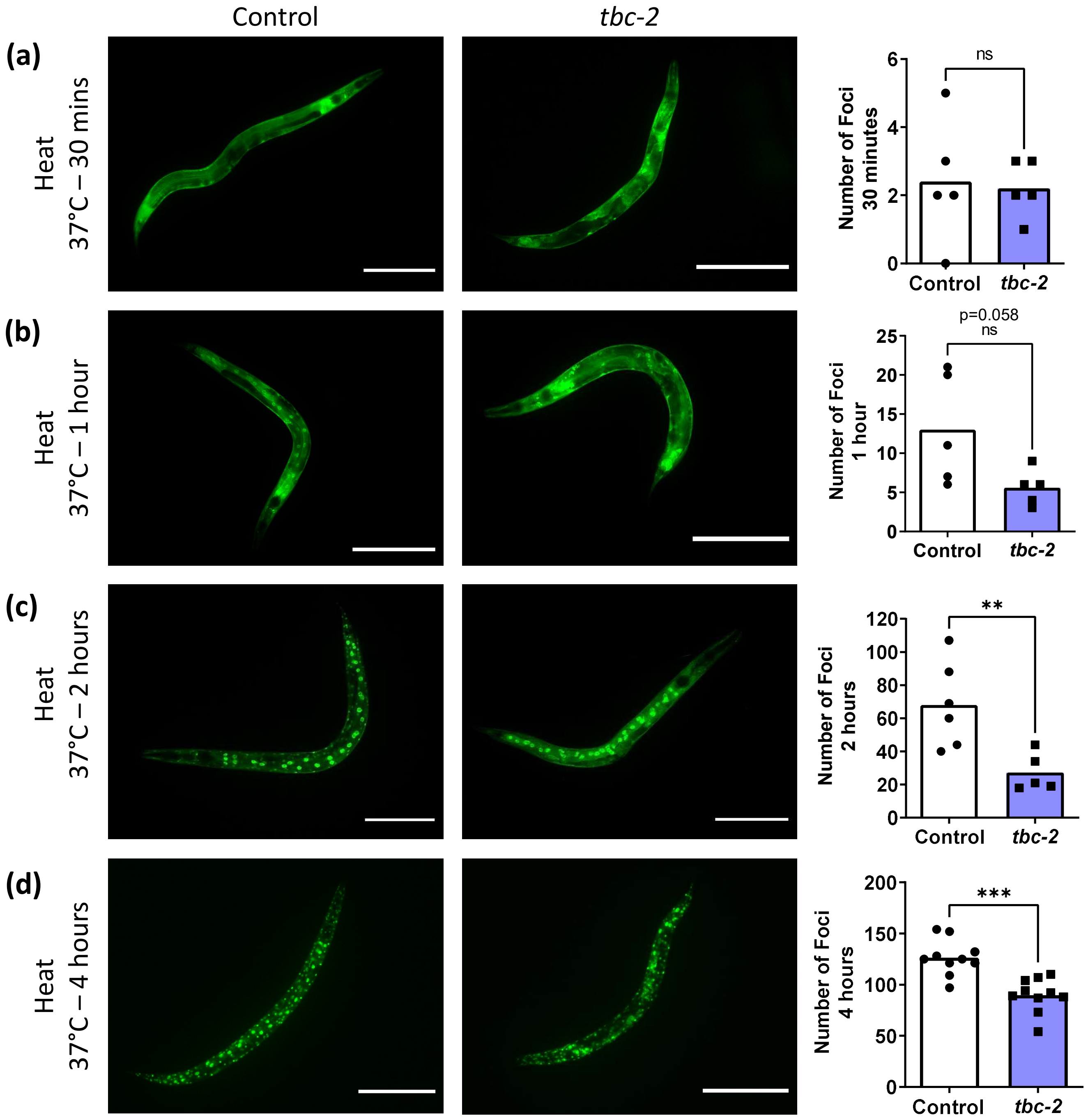

Figure S1 . TBC-2 contributes to the nuclear localization of DAF-16 in response to heat stress. The translational fusion strain *zIs356*[*daf-16p*::*daf-16::GFP*] was used to examine the effect of disrupting *tbc-2* on the nuclear localization of DAF-16 during heat stress. After 30 minutes of heat stress at 37°C, there was minimal nuclear localization of DAF-16, which was equivalent in a wild-type and *tbc-2* mutant background **(a)**. The nuclear localization of DAF-16 increased with the duration of the heat stress. At 1 hour, there was a trend towards decreased nuclear localization of DAF-16 in *tbc-2* mutants **(b).** At 2 hours **(c)** and 4 hours **(d)**, *tbc-2* mutants exhibited significantly less nuclear localization of DAF-16 than control worms. This indicates that TBC-2 is required for the proper nuclear localization of DAF-16 during heat stress. For 30-minute, 1-hour and 2-hour time point, one biological replicate with 5-6 worms per group was performed. The 4-hour time point included three biological replicates. Scale bars indicate 300 µm. Statistical significance was assessed using a t-test. $^{**}p<0.01$, $^{***}p<0.001$.

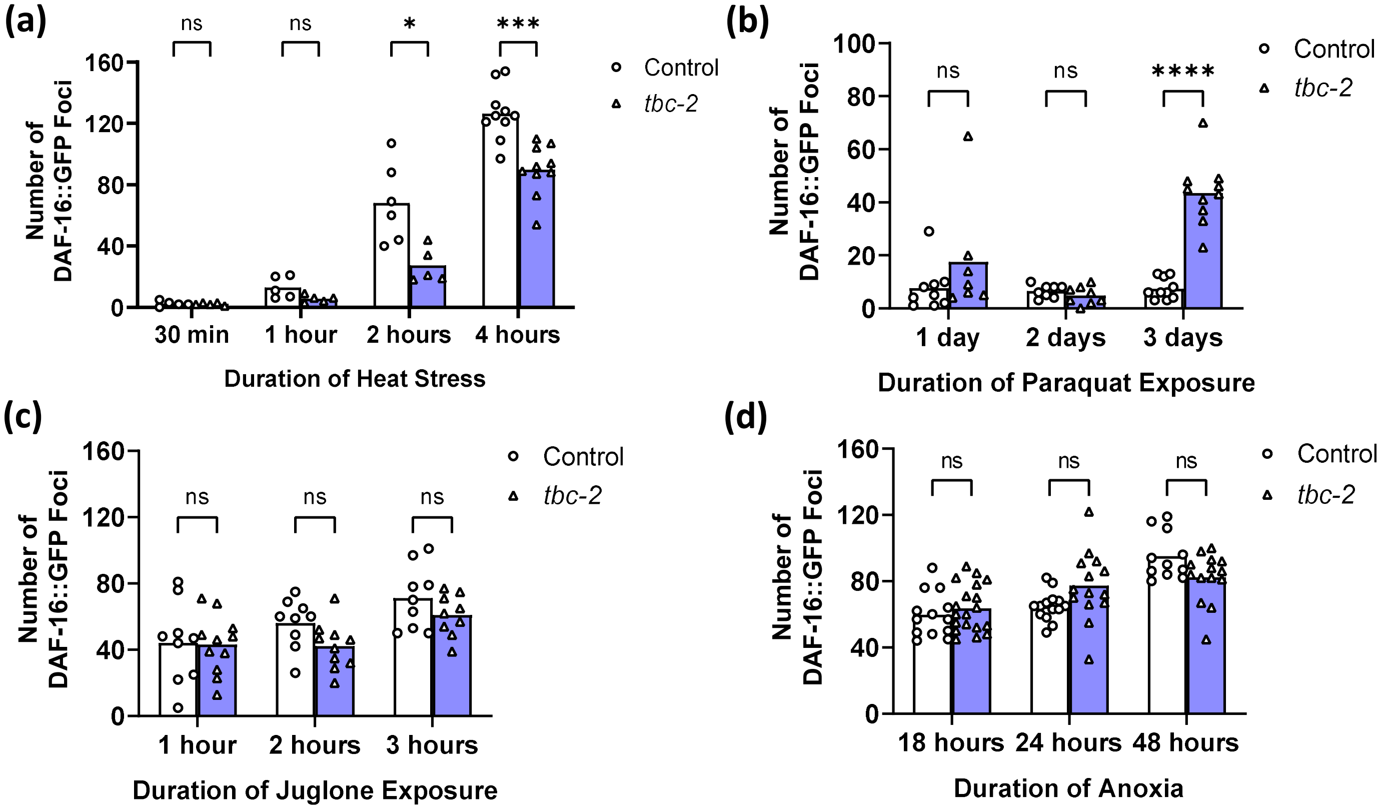

Figure S2 . Time course of nuclear localization of DAF-16 in response to external stressors in wild-type and *tbc-2* mutant background. The translational fusion strain *zIs356*[*daf-16p*::*daf-16::GFP*] was used to examine the effect of disrupting *tbc-2* on the nuclear localization of DAF-16 during heat stress **(a)**, chronic oxidative stress (4 mM paraquat) **(b)**, acute oxidative stress (300 µM juglone) **(c)** or anoxia **(d)**. The wild-type background (control) is indicated by white bars, while the *tbc-2* deletion background is indicated by blue bars. For heat stress, three biological replicates were performed at 4 hours and one biological replicate for the other time points. For paraquat-mediated oxidative stress, three biological replicates were performed at 72 hours and two biological replicates at 24 and 48 hours. For juglone-mediated oxidative stress and anoxia, three biological replicates were performed at each time point. The data for panel (a) is reproduced from Figure S1. The data for the 3-day time point in panel (b), the 3-hour time point in panel (c) and the 48-hour time point for panel (d) are from Figure 1. Statistical significance was assessed using a mixed-effects analysis and Šidák’s multiple comparisons test . *p<0.05, ***p<0.001, ****p<0.0001.

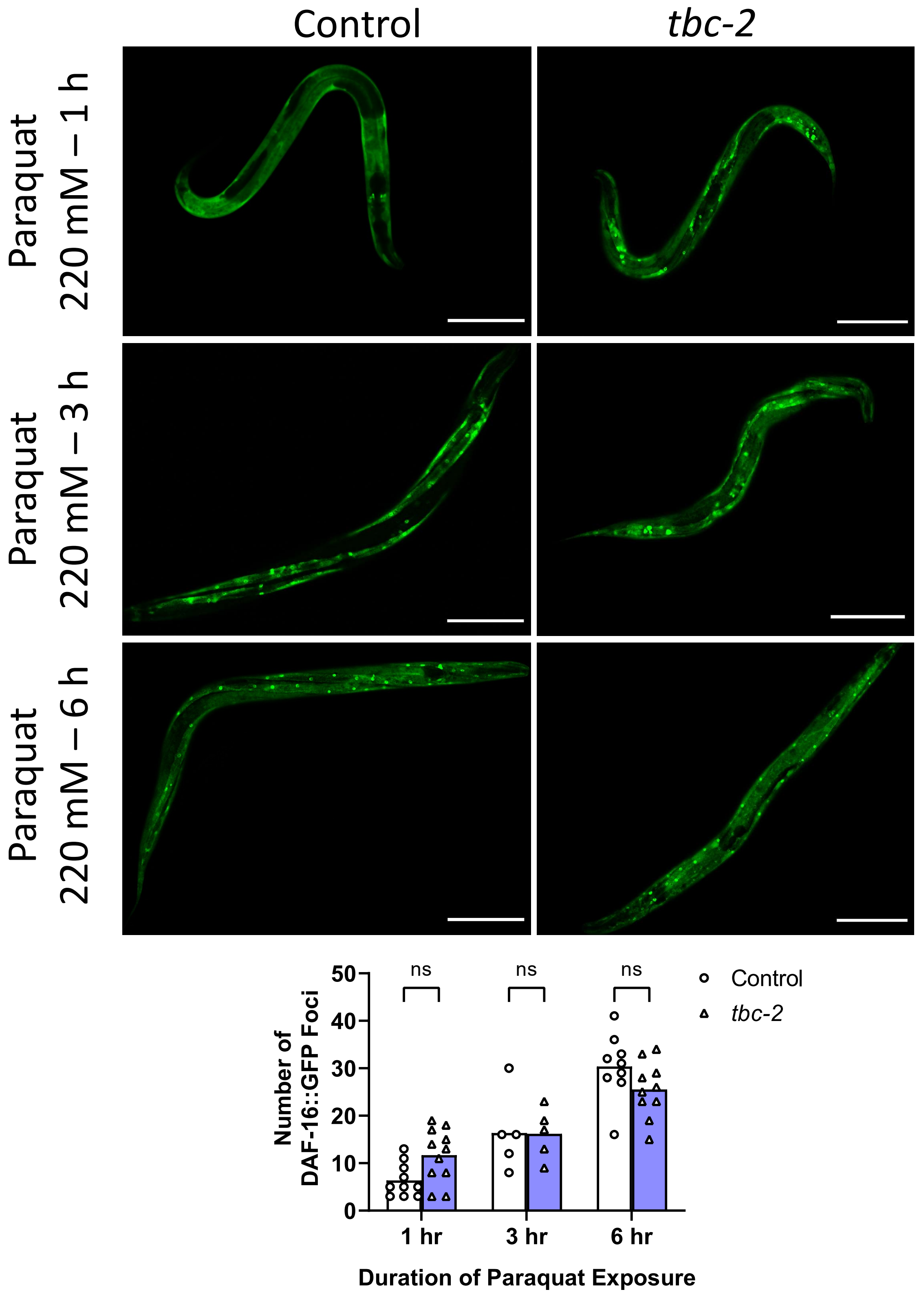

Figure S3. Loss of TBC-2 does not affect nuclear localization of DAF-16 in response to acute paraquat-mediated oxidative stress. Nuclear localization of DAF-16 was examined following exposure to an acute, high dose of paraquat-mediated oxidative stress (220 mM paraquat) using *zIs356*[*daf-16p*::*daf-16::GFP*] worms. At each time point, nuclear localization of DAF-16 was equivalent in wild-type and *tbc-2* mutant backgrounds. Scale bars indicate 100 µm. N=5-11 animals per group. Statistical significance was assessed using mixed-effects analysis and Šidák's multiple comparisons test. **p<0.01, ***p<0.001.

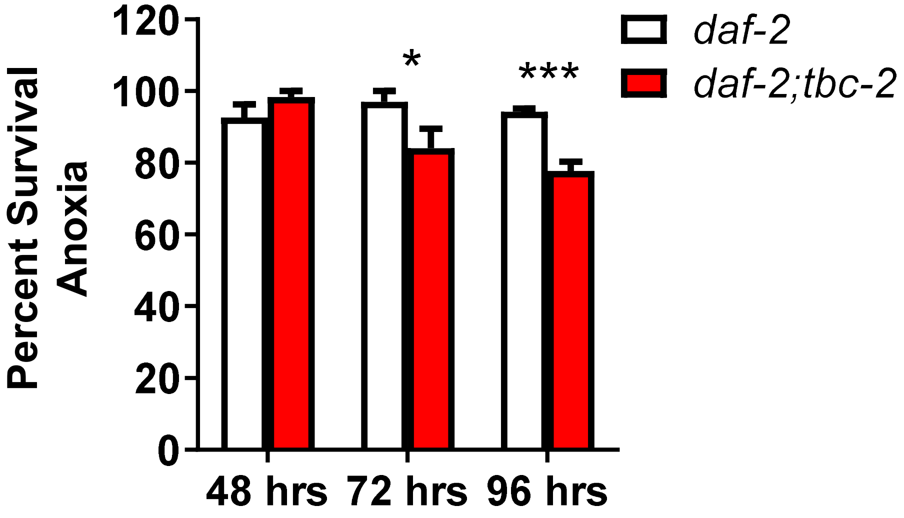

Figure S4. Loss of TBC-2 decreases resistance to anoxia in *daf-2* worms. *daf-2* and *daf-2;tbc-2* worms were exposed to anoxia for 48, 72 or 96 hours. At each time point, *daf-2* worms exhibited close to 100% survival. At the 72- and 96-hour time points, *daf-2;tbc-2* worms had significantly decreased survival compared to *daf-2* mutants. Data from the 48- and 72-hour time points is repeated from Figure 4. Statistical significance was assessed using a two-way ANOVA and Šidák's multiple comparisons test. *$p<0.05$, ***$p<0.001$.

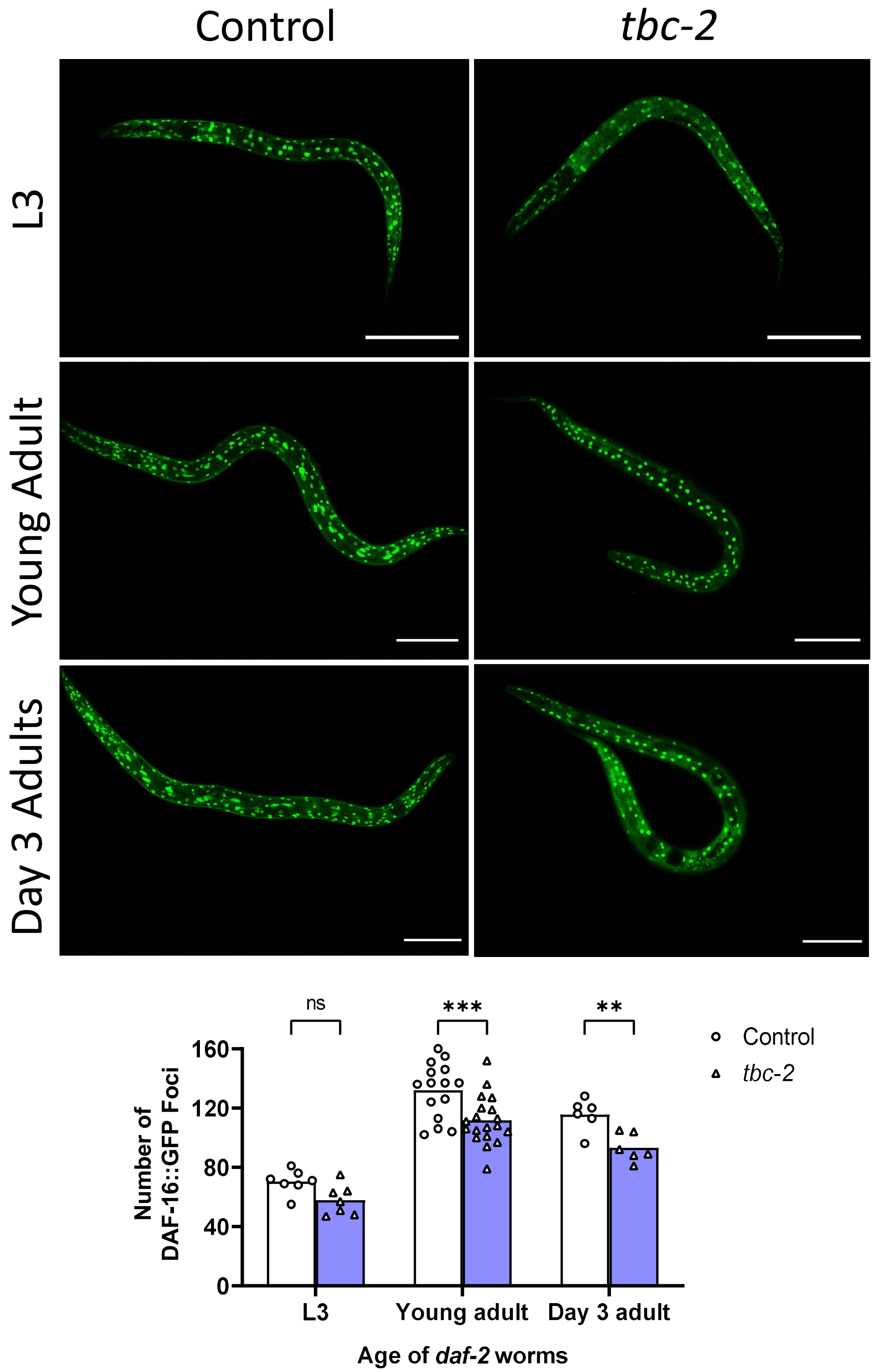

Figure S5. Loss of *tbc-2* decreases nuclear localization of DAF-16 in *daf-2* mutants. The nuclear localization of DAF-16 in *daf-2* mutants was monitored using *zIs356*[*daf-16p*::*daf-16::GFP*] worms. At each time point examined, the disruption of *tbc-2* resulted in a decreased number of DAF-16::GFP foci. This indicates that TBC-2 is required for the full nuclear localization of DAF-16 in *daf-2* worms. Scale bars indicate 100 µm. N=6-15 animals per group. Statistical significance was assessed using mixed-effects analysis and Šidák's multiple comparisons test. $^{**}p<0.01$, $^{***}p<0.001$.

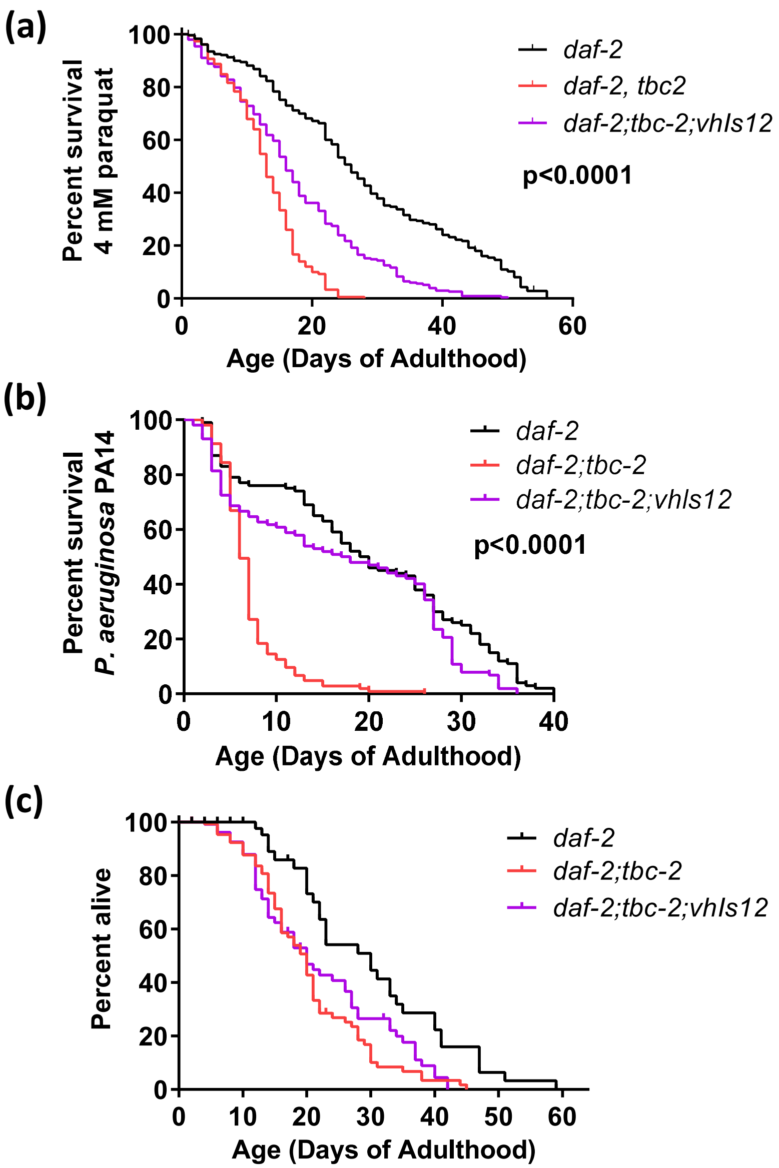

Figure S6. Intestinal expression of wild-type TBC-2 partially rescues decreased stress resistance in *tbc-2* mutants. To examine the ability of intestinally-expressed TBC-2 to rescue deficits in stress resistance and lifespan resulting from disruption of *tbc-2,* we crossed *daf-2;tbc-2* mutants to worms expressing TBC-2 linked to GFP under the intestinal promoter *vha-6* (*vhIs12[vha-6p::GFP::TBC-2]* worms). Expression of TBC-2 in the intestine increased resistance to oxidative stress **(a)** and bacterial pathogen stress **(b)**. There was also a trend towards increased lifespan in *daf-2;tbc-2;vhIs12* worms compared to *daf-2;tbc-2* worms **(c)**. Three biological replicates were performed. Statistical significance was assessed using the log-rank test. p-value shown indicates the significance of difference between *daf-2;tbc-2* (red line) and *daf-2;tbc-2;vhIs12* (purple line).

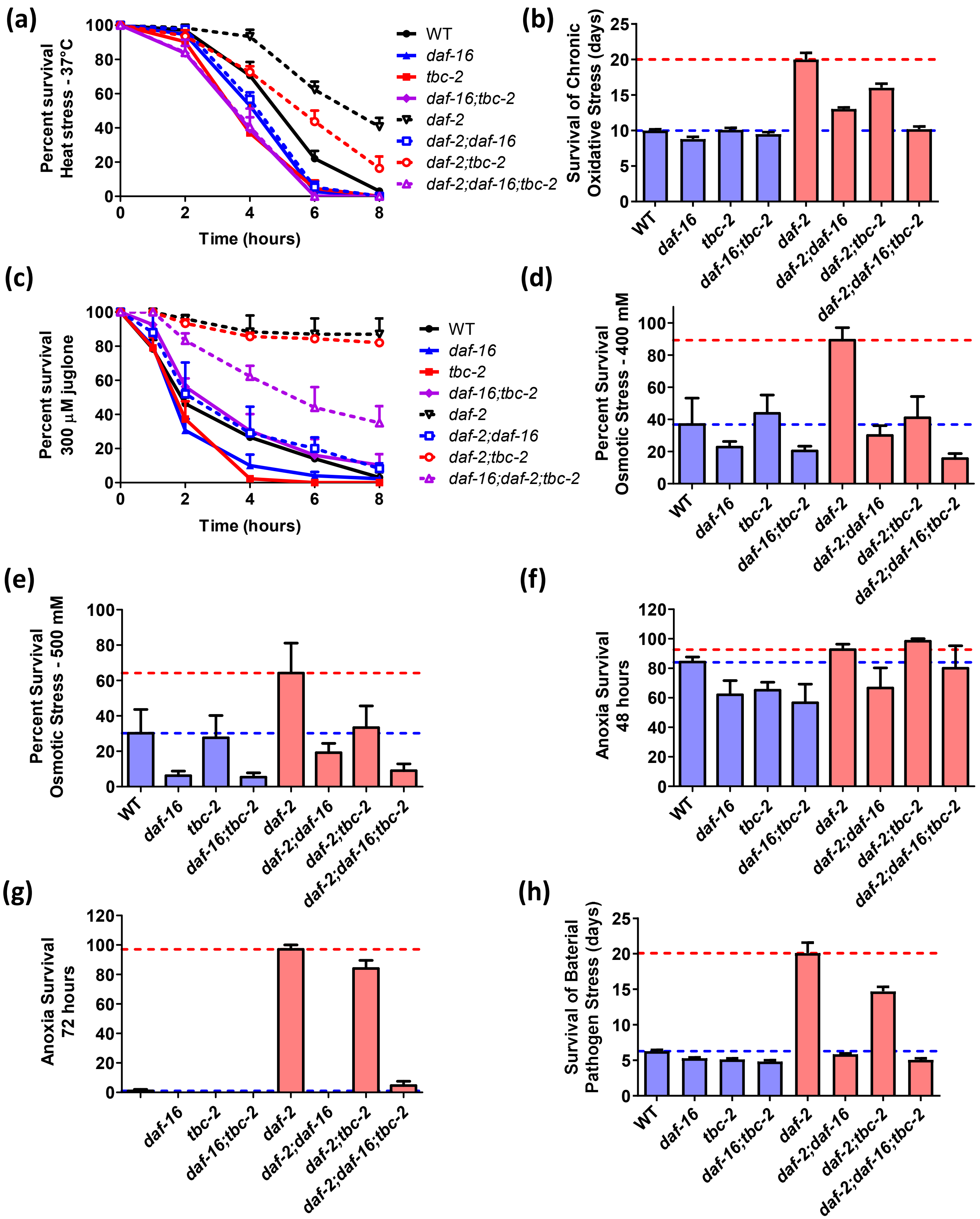

Figure S7. Comparison of stress resistance results across all genotypes. Resistance to heat stress at 37°C **(a)**. Resistance to chronic oxidative stress with 4 mM paraquat **(b)**. Resistance to acute oxidative stress with 300 µM juglone **(c)**. Resistance to osmotic stress with 400 mM NaCl **(d)** or 500 mM NaCl (**e**). Resistance to 48 hours **(f)** or 72 hours **(g)** of anoxia. Resistance to bacterial pathogen stress **(h)**. In panels (b), (d) and (e)-(h), blue dotted line indicates wild-type and red dotted line indicates *daf-2.* The panels from this figure were generated from data from Figures 3 and 4. Statistical analysis can be found with the main figures.

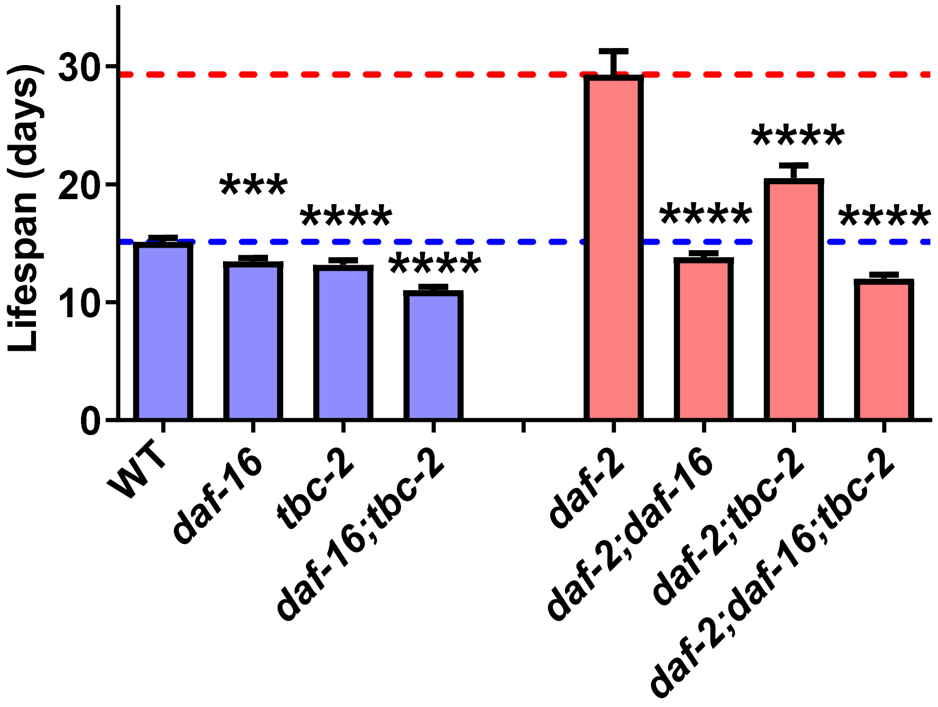

Figure S8. Comparison of lifespan across all genotypes. In a wild-type background (blue bars) disruption of *daf-16* or *tbc-2* result in a similar decrease in lifespan, while *daf-16;tbc-2* double mutants have shorter lifespan than either single mutant. In a *daf-2* background (red bars), loss of *daf-16* decreases lifespan to a greater extent than loss of *tbc-2. daf-2;daf-16;tbc-2* triple mutants have a shorter lifespan than either double mutant. Three biological replicates were performed. Error bars indicate standard error of the mean. Data is displayed as a Kaplan-Meyer survival plot in Figure 6. Statistical significance was determined using a one-way ANOVA and Dunnett's multiple comparison test. Stars show significance of difference from wild-type (blue bars) or *daf-2* (red bars). ***$p<0.001$.
