# Supplementary material for: Endosomal trafficking protein TBC‐2 modulates stress resistance and lifespan through DAF‐16‐dependent and independent mechanisms
Source: Aging Cell. 2023 Feb 15;22(3):e13762. doi: 10.1111/acel.13762 (PMC10014066; doi:10.1111/acel.13762)
Supplement: Supplementary file 2 — TableS1‐S4 [file ACEL-22-e13762-s001.pdf]

Table S1. Significance of difference for comparisons between pairs of specific genotypes

	Heat stress	Paraquat	Juglone	Osmotic 400 mM	Osmotic 500 mM	Anoxia 48 hours	Anoxia 72 hours	Anoxia 96 hours	Bacterial Pathogens	Lifespan
**Effect of *tbc-2* deletion in wild-type background**										
WT versus *tbc-2*	**<0.0001**	0.1002	**0.0046**	0.8667	0.9766	**0.0262**	0.352		**<0.0001**	**0.0011**
daf-2 versus *daf-2;tbc-2*	**<0.0001**	**<0.0001**	0.1898	**0.0019**	0.1229	0.916	0.0568	**0.0007**	**<0.0001**	**<0.0001**
**Effect of *tbc-2* deletion in *daf-16* mutant background**										
daf-16 versus *daf-16;tbc-2*	**0.0396**	0.2033	**<0.0001**	0.9832	0.9978	0.8884	0.9999		**0.024**	**<0.0001**
daf-2;daf-16 vs *daf-2;daf-16;tbc-2*	**0.0097**	**<0.0001**	**<0.0001**	0.4287	0.768	0.6295	0.6001		**0.0383**	**0.0003**
	**	**	**						**	**

**the significance level for log rank test is set at 0.00833 to correct for multiple comparisons
Significant differences are indicated by green text.

Table S3. Summary of the effect of *tbc-2* deletion on nuclear localization of DAF-16, stress resistance and lifespan.

	**Effect of *tbc-2* on Nuclear Localization of DAF-16**	**Effect of *tbc-2* in WT background**	**Effect of *tbc-2* in *daf-2* background**	**Effect of *tbc-2* in *daf-16* background**	**Effect of *tbc-2* in *daf-2;daf-16* background**		**Effect of *daf-16* in WT background**	**Effect of *daf-16* in *daf-2* background**
**Heat Stress**	Decreased	Decreased	Decreased	No effect	No effect		Decreased	Decreased
**Chronic Oxidative Stress**	Increased	No effect	Decreased	No effect	Decreased		Decreased	Decreased
**Acute Oxidative Stress**	No effect	Decreased	No effect	Increased	Increased		Decreased	Decreased
**Osmotic Stress**	Increased	No effect	Decreased	No effect	No effect		Decreased	Decreased
**Anoxia**	Decreased	Decreased	Decreased	No effect	No effect		Decreased	Decreased
**Bacterial Pathogen Stress**	Decreased	Decreased	Decreased	No effect	No effect		Decreased	Decreased
**Lifespan**		Decreased	Decreased	Decreased	Decreased		Decreased	Decreased

Table S4. DAF-16-dependent versus DAF-16-indpendent effects of TBC-2 deletion

	Heat stress	Paraquat	Juglone	Osmotic 400 mM	Osmotic 500 mM	Anoxia 48 hours	Anoxia 72 hours	Bacterial Pathogens	Lifespan
Does *tbc-2* mutation worsen phenotype in *daf-16 mutants?*	No	No	No	No	No	No	No	No	Yes
Does *tbc-2* mutation worsen phenotype in *daf-2;daf-16 mutants?*	No	Yes	No	No	No	No	No	No	Yes
Is the phenotype of *daf-16* mutants equal to or worse than *tbc-2* mutants?	Yes	Yes	Yes	Yes	Yes	Yes	Yes	Yes	Yes
Is the phenotype of *daf-2;daf-16* mutants equal to or worse than *daf-2;tbc-2* mutants?	Yes	Yes	Yes	Yes	Yes	Yes	Yes	Yes	Yes
Results consistent with DAF-16-dependent effect of *tbc-2* mutation	Yes	Yes	Yes	Yes	Yes	Yes	Yes	Yes	No
